# Associations of MDM2 rs2279744 and TP53 rs1042522 polymorphisms with cervical cancer risk: A meta-analysis and systematic review

**DOI:** 10.3389/fonc.2022.973077

**Published:** 2022-08-19

**Authors:** Meijia Yu, Qin Zhang, Xia Zhao

**Affiliations:** ^1^ Department of Gynecology and Obstetrics, Development and Related Disease of Women and Children Key Laboratory of Sichuan Province, Key Laboratory of Birth Defects and Related Diseases of Women and Children, Ministry of Education, West China Second Hospital, Sichuan University, Chengdu, China; ^2^ Department of Obstetrics and Gynecology, The First Hospital of Affiliated to Army Medical University, Chongqing, China; ^3^ Gynecological Cancer Center, Chongqing University Cancer Hospital, Chongqing Cancer Research Institute, Chongqing Cancer Hospital, Chongqing, China

**Keywords:** murine double minute 2, TP53, polymorphism, SNP309T>G, Arg72Pro

## Abstract

**Background:**

Although the association between MDM2 rs2279744 and TP53 rs1042522 polymorphisms and cervical cancer has been reported, the results of its correlation were contradictory. Thus, we conducted a meta-analysis to precisely verify the relationships between MDM2 rs2279744 and TP53 rs1042522 polymorphisms and cervical cancer.

**Methods:**

We thoroughly searched the PubMed, Web of Science, Embase, and Scopus databases for all potential articles from inception to June 2022 and used R Version 4.1.2 and STATA software 12.0 for the meta-analysis. The odds ratios (ORs), 95% confidence intervals (CIs) and 95% prediction intervals (PIs) were calculated to evaluate the associations. Subgroup analyses stratified by ethnicity, source of control, quality score and adjustment were further conducted to assess the relationship between MDM2 rs2279744 and TP53 rs1042522 polymorphisms and cervical cancer.

**Results:**

A total of 30 case-control studies involving 5025 cases and 6680 controls were included. All the included studies were population-based or hospital-based studies. The overall analysis showed that MDM2 rs2279744 polymorphism was closely related to an increased risk of cervical cancer in the recessive model (GG vs GT + TT: OR = 1.602, 95% CI: 1.077-2.383, P = 0.020) and homozygote model (GG vs TT: OR = 1.469, 95% CI: 1.031-2.095, P = 0.033, 95% PI: 0.516-4.184). A significant correlation between TP53 rs1042522 polymorphism and cervical cancer was observed in two models (CC + CG vs GG: OR = 1.759, 95% CI: 1.192-2.596, P = 0.004, 95% PI: 0.474-6.533; GG vs CC: OR = 2.442, 95% CI: 1.433-4.162, P = 0.001, 95% PI: 0.456-13.071).

**Conclusions:**

This meta-analysis revealed that MDM2 SNP309T>G and TP53 rs1042522 C>G polymorphisms were associated with the increased risk of cervical cancer.

## Introduction

Cervical cancer is the second most common gynecological cancer worldwide, with an approximately 570,000 new cases and 311,000 deaths occurring for 2018 worldwide (1, 2). Among the less developed countries, the prevalence and mortality of cervical cancer account for 85% and 87% of the world respectively (3). The persistence of oncogenic human papilloma virus (HPV) infection is the main cause of cervical cancer and its precancerous lesions (4, 5). Nevertheless, most HPV infections are temporary, and 95% of infections are cleared by hosts within 2 years, only 1% progress and develop cervical cancer (6). Many studies have demonstrated that simple HPV infection is not sufficient to cause tumorigenesis and the development of tumors is triggered by the combination of environmental stimuli and human genetic factors (7, 8). Previous studies showed that the heritability accounted for 27% of the total risk of cervical cancer and single nucleotide polymorphisms (SNPs) accounted for about 90% of the genetic variation in the human genome (7, 9).

The TP53 gene, encoding tumor protein 53 (p53), has been well documented for its involvement in DNA damage repair and control of cell cycle (10). In addition, TP53 plays an important role in maintaining genome stability and regulating cell growth and proliferation (11). TP53 mutations can lead to dysfunction and inhibit its DNA damage repair, and somatic TP53 mutations occur in almost every type of cancer (12). Murine double minute 2 (MDM2) gene is a negative regulator of TP53, which can co-regulate cell cycle with TP53 through multiple pathways, thus maintaining genome stability (13). A SNP in the promoter region of MDM2 (SNP309T>G, rs2279744) is capable of leading the abnormal amplification of MDM2 or the enhancement of its expression level, thus resulting in the inactivation of TP53 (14). TP53 rs1042522 polymorphism, a non-synonymous polymorphism causing the substitution of proline for arginine (Arg72Pro) at codon 72, is one of the most common polymorphisms of TP53 and the functional inactivation of TP53 plays a significant role in the occurrence and development of cervical cancer (15, 16). MDM2 rs2279744 and TP53 rs1042522 polymorphisms affect the function of TP53 directly or indirectly by increasing protein expression of MDM2, thereby accelerating the tumorigenesis (17, 18).

Therefore, it is worth to explore the association between MDM2 rs2279744 and TP53 rs1042522 polymorphisms and susceptibility to cervical cancer. However, the reported results are contradictory and ambiguous (19–21). Additionally, several studies have revealed gene-gene interactions between MDM2 rs2279744 and TP53 rs1042522 polymorphisms in a variety of cancers (22–24). Nevertheless, there was no study explicitly reporting an interaction between MDM2 rs2279744 and TP53 rs1042522 polymorphisms in cervical cancer. Consequently, the present study has been designed to estimate whether MDM2 rs2279744 or TP53 rs1042522 polymorphism confers risk to cervical cancer susceptibility and provide support for future research of gene-gene interaction in cervical cancer.

## Methods

### Search strategy

Our study was analyzed and reported according to the Preferred Reporting Items for Systematic Reviews and Meta-Analyses (PRISMA) (25). We thoroughly searched the PubMed, Web of Science, Embase, and Scopus databases for all potential articles from inception to June 2022, using the following search items: (“Murine double minute 2”, “MDM2”, “Tumor protein 53”, “TP53”, or “P53”) AND (“SNP309”, “T309G”, “rs2279744”, “Arg72Pro”, “codon 72 Arg”, “R72P”, or “rs1042522”) AND (“genetic”, “variant”, “polymorphism”, “mutation” or “SNP”) AND (“cervical cancer”, “cervical carcinoma”, “neoplasia”, or “cervix”). References within the identified articles were manually examined to identify other potentially eligible studies.

### Inclusion and exclusion criteria

Studies should meet the following inclusion criteria: (1) study investigating the association of MDM2 rs2279744 or TP53 rs1042522 polymorphism with cervical cancer; (2) meet case-control design; (3) containing available allele or genotype information to estimate an odds ratio (OR) and 95% confidence interval (CI); and (4) results include human subjects. Accordingly, the exclusion criteria were as follows: (1) studies with duplicate data; (2) studies in which subjects were not human; (3) Genome-wide association studies (GWASs) or targeted gene association studies; and (4) conference abstracts, reviews, case reports, and meta-analyses.

### Data extraction and quality assessment

Two independent researchers screened the literature and extracted all needed information from the included studies. All disagreements were resolved by discussion with a third investigator. The following information was extracted from each article using the predesigned data-collection form: name of first author, year of publication, country, ethnicity, sample size, genotyping method, source of control, genotype frequency of investigated SNPs, effect allele, P value for Hardy-Weinberg equilibrium in the control and adjustments. Case-control studies were assessed using the Newcastle-Ottawa scale (NOS) (26) consisting of three domains: (i) selection of subjects, (ii) comparability of groups, and (iii) assessment of outcome. A score of 0-9 was allocated to each relevant study. While the NOS has no established thresholds, we considered the quality of each study as low (0-3 score), medium (4-6 score), or high (7-9 score) (27).

### Statistical analysis

The association between MDM2 rs2279744 or TP53 rs1042522 polymorphism and cervical cancer were estimated by the ORs and their 95% CIs in the five genetic models: dominant model (rs2279744: GG + GT vs TT; rs1042522: CC + CG vs GG or GG + GC vs CC), recessive model (rs2279744: GG vs GT + TT; rs1042522: CC vs CG + GG or GG vs GC + CC), heterozygote model (rs2279744: GT vs TT; rs1042522: CG vs GG or GC vs CC), homozygote model (rs2279744: GG vs TT; rs1042522: CC vs GG or GG vs CC) and allele model (rs2279744: G vs T; rs1042522: C vs G). Because the number of included studies was small and sampled population varied between the studies, random-effects meta-analyses were conducted with the DerSimonian-Laird estimate of heterogeneity (Tau^2^) and the Hartung-Knapp adjustment to calculate ORs with 95% CIs and 95% prediction intervals (PIs). The degree of heterogeneity was assessed with Q, I^2^ and the PI (25). The trim-and-fill method was used to test and adjust for publication bias (28). Sensitivity analysis was conducted to explore the possible sources of heterogeneity. Statistical analyses were performed with R Version 4.1.2 (*meta* package, *metagen* function) and STATA Version 12.0 (StataCorp, College Station, TX, USA). All the P-values are two-sided.

### Trial sequential analysis

Trial Sequential Analysis (TSA) was performed by the TSA v0.9.5.10 Beta software to validate whether the results of the meta-analysis present a definite conclusion (29). Our study set the zero event handing at 1, the type 1 error α 0.05, power 80% and 95% CI to evaluate required information size and the trial sequential monitoring boundary. The number of people with the effect allele was inputted into the intervention group, the number of people with the non-effect allele was inputted into the control group, the number of cervical cancer patients was inputted into events, and the sum of cervical cancer and control subjects was inputted into the total number. According Wacholder et al. (30), an OR value of 1.5 is a reasonable value for the relationship between genes and disease. Hence, our study set the OR value of the correlation between MDM2 rs2279744 polymorphism and cervical cancer as the reciprocal of 1.5 (i.e., 0.67). A review of the past literature indicated that the OR of the association between TP53 rs1042522 polymorphism and cervical cancer was 1.3 in the allele model (31). Consequently, the OR value of TP53 rs1042522 polymorphism and cervical cancer was set at 1.3. Additionally, the 1000 Genome database was used as reference for the minor allele frequency, which is 0.37 for rs2279744 and 0.46 for rs1042522.

## Results

### Literature search and study characteristics

**Figure 1:**
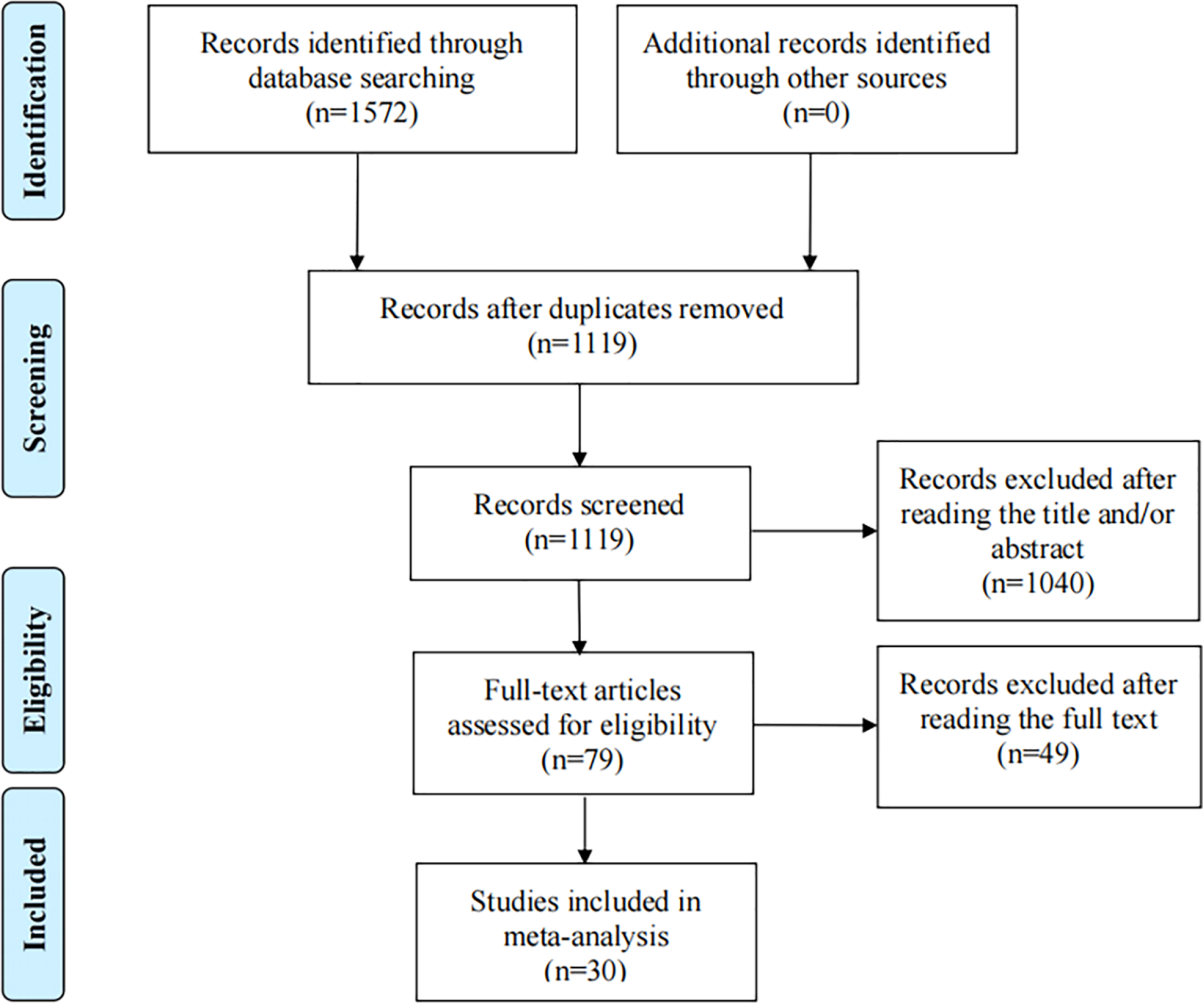
Flow diagram of the process of selection of articles.

Depending on the search strategy, 30 studies with 5025 cases and 6680 controls (32–61) were included in this meta-analysis. **Figure 1** shows the flow diagram of the selection process. Of these, 9 articles reported the results of MDM2 rs2279744 polymorphism and 23 articles focused on the TP53 rs1042522 polymorphism. 2 articles presented the association between cervical cancer and MDM2 rs2279744 and TP53 rs1042522 polymorphisms. **Tables 1**, **2** summarize the extracted data. 17 studies scored ≥ 7 on the NOS, which indicated that these studies were of high quality. 13 studies scored 4-6, indicating moderate quality. **Supplementary Files 1** showed the NOS score for each study.

**Table 1:** Characteristics of the studies of MDM2 rs2279744 polymorphism included in the meta-analysis.

First author	Year	Country	Ethnicity	Numbers		Genotyping method	Source of controls	Genotype			EA	P (HWE) in control	Adjustments	Quality Score
				Cases	Controls			GG	GT	TT				
Guo	2016	China	Asian	180	182	PCR-RFLP	PB	209	66	87	G	>0.05	Age, sex, cigarette smoking, alcohol consumption and family history	7
Tantengco	2019	Philippine	Asian	28	21	PCR Sequencing	HB	5	19	20	G	>0.05	NA	6
Jiang	2011	China	Asian	105	140	PCR-RFLP	HB	64	134	47	G	NA	Age, smoking, drinking and family history	6
Vargas-Torres	2014	Brazil	Latino	293	184	PCR-RFLP	PB	43	186	248	G	0.644	NA	8
Al-Harbi	2017	Saudi Arabia	Asian	232	313	PCR Sequencing	PB	132	260	153	G	0.885	NA	7
Alsbeih	2013	Saudi Arabia	Asian	100	100	PCR Sequencing	PB	53	90	57	G	>0.05	Age	7
Roszak	2015	Poland	European	456	481	PCR primer pairing	HB	153	408	376	G	0.37	Age, pregnancy, oral contraceptive use, tobacco smoking, and menopausal status	6
Singhal	2013	India	Asian	182	182	PCR-RFLP	HB	67	126	171	G	>0.05	Age and ethnicity	7
Meissner	2007	Brazil	Latino	70	100	PIRA-PCR assay	PB	22	89	61	G	>0.05	Age and ethnicity	7

EA, effect allele; HWE, Hardy-Weinberg equilibrium; PCR-RFLP, polymerase chain reaction-restriction fragment length polymorphism; PB, population-based study; HB, hospital-based study; PIRA, primer-introduced restriction analysis; NA, not available.

**Table 2:** Characteristics of the studies of TP53 rs1042522 polymorphism included in the meta-analysis.

First author	Year	Country	Ethnicity	Numbers		Genotyping method	Source of control	Genotype			EA	P (HWE) in control	Adjustments	Quality Score
				Cases	Controls			CC	CG	GG				
Yuan	2016	China	Asian	328	568	PCR-RFLP	HB	116	493	287	C	0.08	Age	7
Barbisan	2011	Argentina	Latino	98	123	PCR Sequencing	PB	13	97	111	C	>0.05	Age and HPV	7
Mostaid	2021	Bangladesh	Asian	129	122	PCR-RFLP	HB	39	68	144	C	>0.05	NA	7
Laprano	2014	Brazil	Latino	45	88	PCR-RFLP	HB	17	65	51	C	>0.05	NA	6
Niwa	2004	Japan	Asian	112	442	PCR-CTPP	PB	71	261	222	C	0.92	Age	7
Alsbeih	2013	Saudi Arabia	Asian	100	100	PCR Sequencing	PB	48	110	42	C	0.72	Age	7
Liu	2019	China	Asian	121	118	MAMA-PCR	HB	87	101	41	C	0.74	NA	6
González-Herrera	2014	Mexico	Latino	78	274	PCR-RFLP	PB	18	147	187	C	0.21	NA	8
Roh	2010	Korea	Asian	53	286	PCR Sequencing	PB	NA	NA	182	C	>0.05	NA	7
Ye	2010	China	Asian	500	800	PCR Sequencing	HB	279	771	250	C	>0.05	Age	6
Zhou	2009	China	Asian	404	404	PCR-RFLP	PB	163	404	241	C	0.406	Age, smoking status, menopausal status, family history of cancer and parity	9
Datkhile	2019	India	Asian	350	400	PCR-RFLP	HB	174	394	182	G	NA	NA	6
Santos	2005	Portugal	European	164	145	AS-PCR	PB	20	87	202	G	>0.05	NA	6
Malisic	2013	Serbia	European	49	74	PCR-RFLP	HB	7	42	74	G	>0.05	NA	8
Apu	2020	Bangladesh	Asian	134	102	PCR-RFLP	HB	36	62	129	C	>0.05	NA	6
Ratre	2019	India	Asian	100	100	PCR-RFLP	HB	67	59	74	G	>0.05	NA	7
Singhal	2013	India	Asian	182	182	PCR-RFLP	HB	100	170	94	G	>0.05	Age, ethnic	7
Klug	2001	Peru	Latino	119	127	PCR-RFLP	HB	30	90	126	G	>0.05	Age and HPV	7
Jiang	2010	China	Asian	104	160	PCR-RFLP	PB	70	131	63	G	>0.05	Age, cigarette smoking, alcohol consumption and family history	8
Assoumou	2015	Gabon	African	31	71	PCR Sequencing	PB	15	60	27	G	>0.05	NA	5
Gudleviciene	2006	Lithuania	European	141	97	PCR-RFLP	HB	35	149	54	G	NA	Age	6
Saranath	2002	India	Asian	134	131	PCR Sequencing	HB	53	165	47	G	NA	NA	6
Lee	2004	Korea	Asian	185	345	SNaPshot assay	HB	84	242	204	G	NA	Age, education level, age at first intercourse, and number of children	4

EA, effect allele; HWE, Hardy-Weinberg equilibrium; PCR-RFLP, polymerase chain reaction-restriction fragment length polymorphism; HB, hospital-based study; PB, population-based study; PCR-CTPP, PCR with confronting two-pair primers; MAMA-PCR, mismatch amplification mutation assay PCR; AS-PCR, allele-specific polymerase chain reaction; NA, not available.

### Association between MDM2 rs2279744 polymorphism and cervical cancer susceptibility

#### Overall analysis

**Figure 2:**
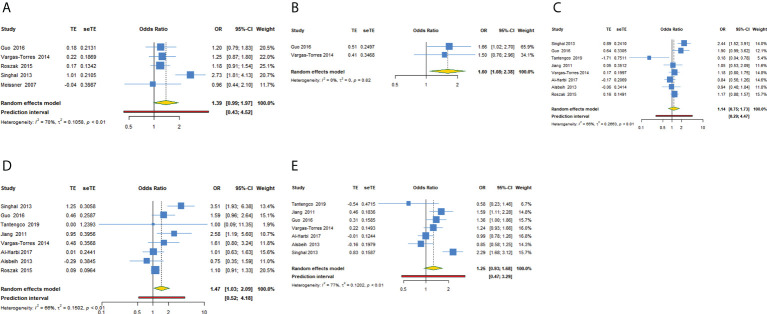
Forest plot of MDM2 rs2279744 polymorphism and cervical cancer susceptibility in five models. **(A)** GG + GT vs TT; **(B)** GG vs GT + TT; **(C)** GT vs TT; **(D)** GG vs TT; **(E)** G vs T.

We investigated the association of MDM2 rs2279744 polymorphism and cervical cancer susceptibility in five genetic models (**Table 3**, **Figures 2A–E**). 2 studies reported data showing the association in recessive model. The pooled OR indicated that MDM2 rs2279744 polymorphism in recessive model was closely related to an increased risk of cervical cancer (OR = 1.602, 95% CI: 1.077-2.383, P = 0.020). A heterogeneity test showed low heterogeneity among 2 studies (I^2^ = 0.0%, Tau^2^ = 0) (**Figure 2B**). The association between MDM2 rs2279744 polymorphism and cervical cancer susceptibility in homozygote model was assayed using data from 8 studies, and we found that MDM2 rs2279744 polymorphism in homozygote model was significantly related to high cervical cancer susceptibility (OR = 1.469, 95% CI: 1.031-2.095, P = 0.033; 95% PI: 0.516-4.184), however, heterogeneity was found to be relatively large (I^2^ = 65.7%, Tau^2^ = 0.1502) (**Figure 2D**). No relevance was observed in the dominant, heterozygote or allele model.

**Table 3:** The association between MDM2 rs2279744 polymorphism and cervical cancer susceptibility.

Outcome and subgroups	Dominant model (GG + GT vs TT)				Recessive model (GG vs GT + TT)				Heterozygote model (GT vs TT)				Homozygote model (GG vs TT)					Allele model (G vs T)			
	No	OR (95% CI)	I^2^, %	95% PI	No	OR (95% CI)	I^2^, %	95% PI	No	OR (95% CI)	I^2^, %	95% PI	No	OR (95% CI)	I^2^, %	95% PI	No		OR (95% CI)	I^2^, %	95% PI
Overall	5	1.393 (0.987-1.968)	70.2	0.429-4.522	2	**1.602 (1.077-2.383)**	0.0	–	8	1.140 (0.751-1.729)	66.1	0.291-4.465	8	**1.469 (1.031-2.095)**	65.7	0.516-4.184	7		1.246 (0.927-1.676)	77.2	0.472-3.294
Ethnicity																					
Asian	2	1.815 (0.812-4.057)	86.7	–	1	**1.657 (1.016-2.703)**	–	–	6	1.079 (0.576-2.021)	75.7	0.130-8.985	6	1.578 (0.969-2.569)	67.9	0.361-6.902	6		1.238 (0.865-1.772)	81.0	0.370-4.145
European	1	1.180 (0.907-1.535)	–	–					1	1.174 (0.876-1.573)	–	–	1	1.099 (0.910-1.328)	–	–					
Other	2	1.192 (0.856-1.661)	0.0	–	1	1.500 (0.760-2.960)	–	–	1	1.180 (0.798-1.745)	–	–	1	1.610 (0.800-3.240)	–	–	1		1.240 (0.925-1.662)	–	–
Source of control																					
PB	3	1.196 (0.923-1.551)	0.0	0.222-6.443	2	**1.602 (1.077-2.383)**	0.0	–	4	1.109 (0.802-1.534)	36.7	0.353-3.481	4	1.208 (0.864-1.687)	22.0	0.435-3.352	4		1.103 (0.906-1.343)	38.3	0.547-2.225
HB	2	1.768 (0.777-4.026)	91.2	–					4	1.002 (0.376-2.668)	79.4	0.011-90.327	4	**1.902 (1.048-3.451)**	81.8	0.173-20.970	3		1.418 (0.670-2.998)	76.5	–
Quality score																					
High	4	1.464 (0.939-2.283)	73.7	0.218-9.816	2	**1.602 (1.077-2.383)**	0.0	–	5	1.328 (0.887-1.988)	70.8	0.337-5.235	5	1.482 (0.895-2.454)	70.7	0.258-8.504	5		1.270 (0.914-1.766)	81.9	0.379-4.259
Moderate	1	1.180 (0.907-1.535)	–	–					3	0.727 (0.249-2.124)	66.7	–	3	1.403 (0.816-2.412)	54.6	0.006-319.575	2		1.054 (0.399-2.785)	74.8	–
Adjustment																					
Yes	4	1.431 (0.914-2.240)	77.2	0.203-10.066	1	**1.657 (1.016-2.703)**	–	–	5	1.420 (0.995-2.026)	58.2	0.468-4.310	5	1.612 (0.949-2.741)	79.1	0.243-10.688	4		1.446 (0.970-2.157)	81.0	0.236-8.856
NR	1	1.250 (0.867-1.803)	–	–	1	1.500 (0.760-2.960)	–	–	3	0.684 (0.251-1.866)	68.9	–	3	1.167 (0.790-1.723)	0.0	0.093-14.599	3		1.039 (0.777-1.391)	34.2	0.057-18.817

#### Subgroup analysis

In subgroup analysis of recessive model conducted by source of control or quality score, we obtained the same significant results in the subgroup of population-based study or high-quality study (OR = 1.602, 95% CI: 1.077-2.383). In subgroup analysis conducted by ethnicity or adjustment, we obtained the same significant results in Asians or studies with adjusted results in recessive model (OR = 1.657, 95% CI: 1.016-2.703). No significant results were observed in subgroup analysis of dominant, heterozygote, homozygote or allele model. **Supplementary Files 2**; **Table 3** showed the subgroup analysis in five models for MDM2 rs2279744 polymorphism.

### Association between TP53 rs1042522 polymorphism and cervical cancer susceptibility

#### Overall analysis

**Figure 3:**
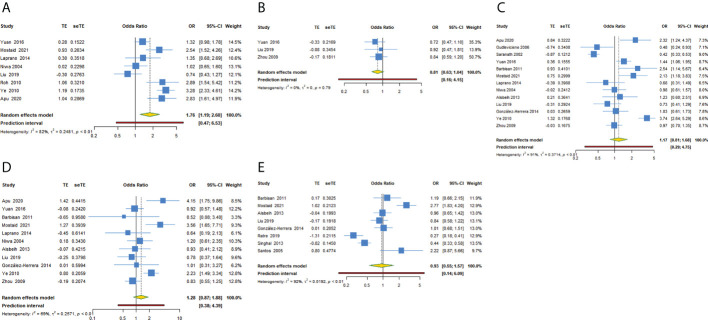
Forest plot of TP53 rs1042522 polymorphism and cervical cancer susceptibility (allele C as the effect allele) in five models. **(A)** CC + CG vs GG; **(B)** CC vs CG + GG; **(C)** CG vs GG; **(D)** CC vs GG; **(E)** C vs G.

**Figure 4:**
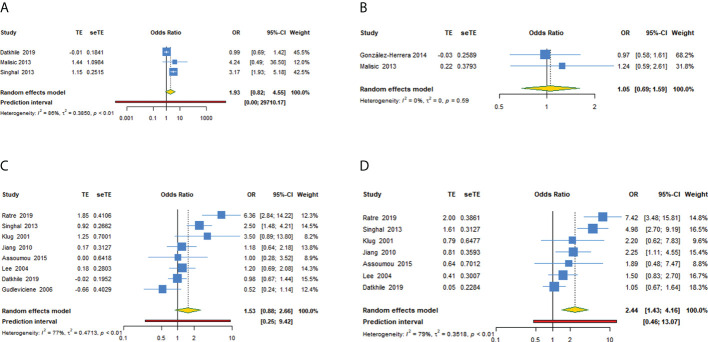
Forest plot of TP53 rs1042522 polymorphism and cervical cancer susceptibility (allele G as the effect allele) in four models. **(A)** GG + GC vs CC; **(B)** GG vs GC + CC; **(C)** GC vs CC; **(D)** GG vs CC.

For TP53 rs1042522 polymorphism and cervical cancer susceptibility, 15 studies reported results showing the allele C as the effect allele, while the G allele was regarded as effect allele in 8 studies. We explored the association between TP53 rs1042522 polymorphism and cervical cancer susceptibility in five genetic models for effect allele C (**Table 4**, **Figures 3A–E**) and four models for effect allele G (**Table 5**, **Figures 4A–D**). In the overall population, we observed a significant relationship with cervical cancer in two models (CC + CG vs GG: OR = 1.759, 95% CI: 1.192-2.596, P = 0.004, 95% PI: 0.474-6.533; GG vs CC: OR = 2.442, 95% CI: 1.433-4.162, P = 0.001, 95% PI: 0.456-13.071). A heterogeneity test showed relatively high heterogeneity among these studies (CC + CG vs GG: I^2^ = 82.3%, Tau^2^ = 0.2481; GG vs CC: I^2^ = 79.2%, Tau^2^ = 0.3518) (**Figure 3A**, **Figure 4D**).

**Table 4:** The association between TP53 rs1042522 polymorphism and cervical cancer susceptibility (allele C as the effect allele).

Outcome and subgroups	Dominant model (CC + CG vs GG)				Recessive model (CC vs CG + GG)				Heterozygote model (CG vs GG)				Homozygote model (CC vs GG)				Allele model (C vs G)			
	No	OR (95% CI)	I2, %	95% PI	No	OR (95% CI)	I2, %	95% PI	No	OR (95% CI)	I2, %	95% PI	No	OR (95% CI)	I2, %	95% PI	No	OR (95% CI)	I2, %	95% PI
Overall	8	**1.759 (1.192-2.596)**	82.3	0.474-6.533	3	0.806 (0.626-1.037)	0.0	0.157-4.148	13	1.168 (0.811-1.681)	91.3	0.287-4.747	11	1.283 (0.874-1.885)	68.7	0.375-4.389	8	0.927 (0.546-1.572)	91.9	0.141-6.089
Ethnicity																				
Asian	7	**1.817 (1.172-2.814)**	84.6	0.407-8.110	3	0.806 (0.626-1.037)	0.0	0.157-4.148	9	1.268 (0.815-1.972)	93.7	0.261-6.173	8	1.429 (0.906-2.255)	76.2	0.317-6.438	5	0.765 (0.354-1.651)	94.7	0.038-15.336
European									1	**0.476 (0.244-0.928)**	–	–					1	2.222 (0.872-5.664)	–	–
Other	1	1.350 (0.678-2.690)	–	–					3	1.192 (0.572-2.481)	64.5	–	3	0.751 (0.349-1.618)	0.0	0.005-108.670	2	1.064 (0.763-1.484)	0.0	–
Source of control																				
PB	2	1.676 (0.605-4.646)	85.6	–	1	0.840 (0.589-1.198)	–	–	5	1.120 (0.845-1.485)	21.3	0.537-2.333	5	0.910 (0.671-1.235)	0.0	0.555-1.494	4	1.075 (0.841-1.372)	0.0	0.628-1.838
HB	6	**1.795 (1.133-2.844)**	84.0	0.371-8.675	2	0.772 (0.538-1.106)	0.0	–	8	1.139 (0.646-2.009)	94.7	0.151-8.565	6	1.610 (0.859-3.020)	77.7	0.196-13.223	4	0.724 (0.271-1.935)	95.9	0.006-84.228
Quality Score																				
High	4	**1.707 (1.050-2.773)**	74.3	0.200-14.539	2	0.789 (0.600-1.035)	0.0	–	7	1.289 (0.997-1.667)	44.4	0.647-2.570	7	1.122 (0.753-1.671)	49.9	0.379-3.317	6	0.845 (0.440-1.621)	93.8	0.079-9.014
Moderate	4	1.782 (0.899-3.533)	87.2	0.077-41.354	1	0.920 (0.468-1.810)	–	–	6	0.983 (0.474-2.036)	95.8	0.072-13.377	4	1.547 (0.668-3.580)	75.7	0.038-63.498	2	1.238 (0.487-3.150)	72.0	–
Adjustment																				
Yes	3	1.654 (0.827-3.307)	90.9	–	2	0.789 (0.600-1.035)	0.0	–	7	1.347 (0.819-2.216)	88.0	0.244-7.447	6	1.136 (0.794-1.625)	65.6	0.416-3.101	3	0.766 (0.420-1.396)	86.7	–
NR	5	**1.836 (1.084-3.110)**	77.0	0.284-11.875	1	0.920 (0.468-1.810)	–	–	6	0.986 (0.569-1.709)	89.5	0.147-5.590	5	1.572 (0.723-3.417)	73.5	0.108-22.898	5	1.042 (0.464-2.340)	93.8	0.047-23.079

**Table 5:** The association between TP53 rs1042522 polymorphism and cervical cancer susceptibility (allele G as the effect allele).

Outcome and subgroups	Dominant model (GG + GC vs CC)				Recessive model (GG vs GC + CC)				Heterozygote model (GC vs CC)				Homozygote model (GG vs CC)			
	No	OR (95% CI)	I^2^, %	95% PI	No	OR (95% CI)	I^2^, %	95% PI	No	OR (95% CI)	I^2^, %	95% PI	No	OR (95% CI)	I^2^, %	95% PI
Overall	3	1.932 (0.821-4.547)	86.5	–	2	1.049 (0.690-1.595)	0.0	–	8	1.534 (0.885-2.658)	76.9	0.250-9.416	7	**2.442 (1.433-4.162)**	79.2	0.456-13.071
Ethnicity																
Asian	2	1.749 (0.560-5.463)	92.8	–					5	1.782 (0.923-3.439)	81.8	0.155-20.436	5	**2.576 (1.259-5.271)**	86.1	0.180-36.817
European	1	4.240 (0.493-36.503)	–	–	1	1.240 (0.590-2.608)	–	–	1	0.518 (0.235-1.141)	–	–				
Other					1	0.970 (0.584-1.611)	–	–	2	1.813 (0.532-6.180)	42.5	–	2	2.051 (0.807-5.213)	0.0	–
Source of control																
PB					1	0.970 (0.584-1.611)	–	–	2	1.143 (0.659-1.983)	0.0	–	2	**2.170 (1.159-4.061)**	0.0	–
HB	3	1.932 (0.821-4.547)	86.5	–	1	1.240 (0.590-2.608)	–	–	6	1.711 (0.830-3.528)	83.2	0.141-20.771	5	**2.589 (1.239-5.410)**	86.1	0.179-37.547
Quality Score																
High	2	**3.213 (1.987-5.196)**	0.0	–	2	1.049 (0.690-1.595)	0	–	4	**2.677 (1.317-5.444)**	73.0	0.133-53.997	4	**3.934 (2.210-7.001)**	53.5	0.428-36.195
Moderate	1	0.990 (0.690-1.420)	–	–					4	0.954 (0.718-1.267)	0.0	0.511-1.779	3	1.231 (0.872-1.739)	0.0	0.132-11.528
Adjustment																
Yes	1	**3.167 (1.934-5.185)**	–	–					5	1.369 (0.749-2.503)	69.6	0.173-10.866	4	**2.509 (1.465-4.298)**	61.8	0.317-19.843
NR	2	1.369 (0.418-4.485)	41.4	–	2	1.049 (0.690-1.595)	0	–	3	1.857 (0.548-6.295)	88.3	–	3	2.431 (0.747-7.915)	89.5	–

#### Subgroup analysis

**Supplementary Files 3** and **Tables 4**, **5** showed the subgroup analysis of each model for TP53 rs1042522 polymorphism. In a subgroup analysis conducted by ethnicity, we obtained significant results in Asians that TP53 rs1042522 polymorphism was associated with an increased the risk of cervical cancer in two models (CC + CG vs GG: OR = 1.817, 95% CI: 1.172-2.814, 95% PI: 0.407-8.110; GG vs CC: OR = 2.576, 95% CI: 1.259-5.271, 95% PI: 0.180-36.817). We also observed that TP53 rs1042522 polymorphism decreased the risk of cervical cancer in the European population in one model (CG vs GG: OR = 0.476, 95% CI: 0.244-0.928).

In a subgroup analysis conducted by source of control, we obtained significant results in hospital-based population in two models (CC + CG vs GG: OR = 1.795, 95% CI: 1.133-2.844, 95% PI: 0.371-8.675; GG vs CC: OR = 2.589, 95% CI: 1.239-5.410, 95% PI: 0.179-37.547) and in population-based studies in one model (GG vs CC: OR = 2.170, 95% CI: 1.159-4.061).

In a subgroup analysis conducted by quality score, we obtained significant results in high-quality studies that TP53 rs1042522 polymorphism increased the risk of cervical cancer in four models (CC + CG vs GG: OR = 1.707, 95% CI: 1.050-2.773, 95% PI: 0.200-14.539; GG + GC vs CC: OR = 3.213, 95% CI: 1.987-5.196; GC vs CC: OR = 2.677, 95% CI: 1.317-5.444, 95% PI: 0.133-53.997; GG vs CC: OR = 3.934, 95% CI: 2.210-7.001, 95% PI: 0.428-36.195). No significant results in low-quality studies were observed in all models.

In a subgroup analysis conducted by adjustment, we found that TP53 rs1042522 polymorphism was associated with an increased susceptibility to cervical cancer in covariates-adjusted studies in two models (GG + GC vs CC: OR = 3.167, 95% CI: 1.934-5.185; GG vs CC: OR = 2.509, 95% CI: 1.465-4.298, 95% PI: 0.317-19.843). We also obtained a significant result that TP53 rs1042522 polymorphism increased the risk of cervical cancer in studies without definite adjustments in one model (CC + CG vs GG: OR = 1.836, 95% CI: 1.084-3.110, 95% PI: 0.284-11.875).

### Sensitivity analysis and publication bias

To assess whether the pooled results were affected by a single study, we conducted a sensitivity analysis by computing the pooled ORs and the corresponding 95% CIs after individual studies were omitted. The results were shown in **Supplementary Files 4**. The removal of any single study did not significantly affect the quantitative results, suggesting that the results were robust and reliable. Trim-and-fill analysis was conducted and 7 funnel plots with imputed studies were obtained, revealing the existence of publication bias in seven models. By comparing the pooled effects before and after the trim-and-fill analysis, we found that the existence of publication bias changed the results in three models (MDM2 rs2279744: GG vs TT; TP53 rs1042522: CC vs CG + GG and GG vs CC). Correction for potential publication bias did not alter the association in four models (TP53 rs1042522: CG vs GG, C vs G, GG + GC vs CC and GG vs GC + CC) (**Table 6**). The funnel plots of all models were in **Supplementary Files 5**.

**Table 6:** Comparison of pooled effects before and after trim-and-fill analysis.

Genetic models	Before trim-and-fill analysis		After trim-and-fill analysis	
	OR	95% CI	OR	95% CI
MDM2 rs2279744				
GG + GT vs TT	1.393	0.987-1.968	1.393	0.987-1.968
GG vs GT + TT	1.602	1.077-2.383	1.602	1.077-2.383
GT vs TT	1.140	0.751-1.729	1.140	0.751-1.729
GG vs TT	1.469	1.031-2.095	**1.149**	**0.785-1.684**
G vs T	1.246	0.927-1.676	1.246	0.927-1.676
TP53 rs1042522				
CC + CG vs GG	1.759	1.192-2.596	1.759	1.192-2.596
CC vs CG + GG	0.806	0.626-1.037	**0.788**	**0.622-0.999**
CG vs GG	1.168	0.811-1.681	0.665	0.413-1.072
CC vs GG	1.283	0.874-1.885	1.283	0.874-1.885
C vs G	0.927	0.546-1.572	0.614	0.355-1.063
GG + GC vs CC	1.932	0.821-4.547	0.990	0.355-2.759
GG vs GC + CC	1.049	0.690-1.595	0.970	0.673-1.397
GC vs CC	1.534	0.885-2.658	1.534	0.885-2.658
GG vs CC	2.442	1.433-4.162	**1.275**	**0.656-2.478**

### Trial sequential analysis results

**Figure 5:**
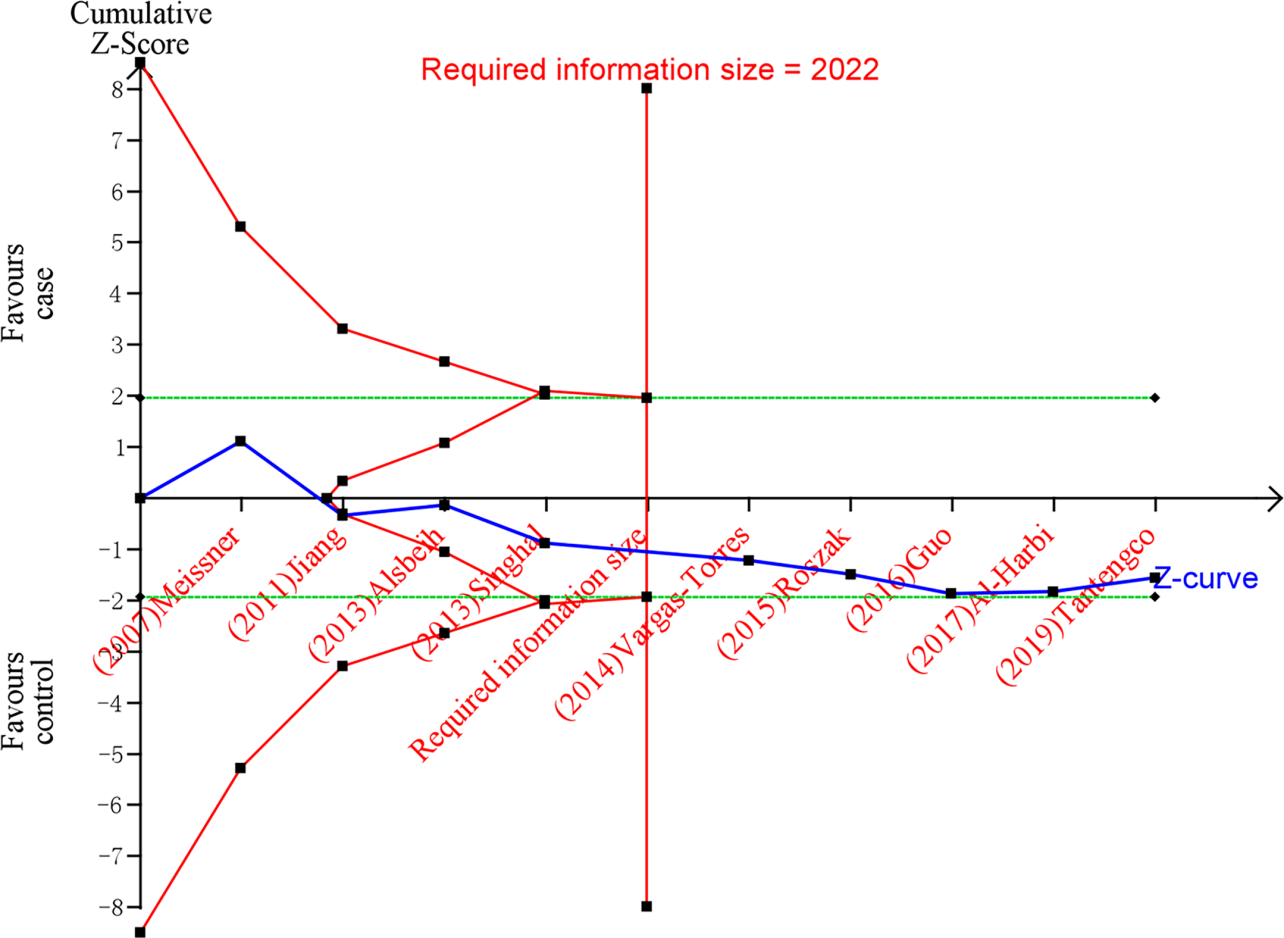
Estimation of the sample size for the relationship between MDM2 rs2279744 polymorphism and cervical cancer. Trial sequential analysis (TSA) is a methodology that includes a sample size calculation for a meta-analysis with the threshold of statistical significance. We performed a TSA using an allele model assumption but replaced the allele count with the sample size (divided by 2). Detailed settings: Significance level = 0.05; power = 0.80; ratio of controls to cases = 1; hypothetical proportion of effect allele in control = 0.37; least extreme OR to be detected = 1.5; I^2^ (heterogeneity) = 74%.

**Figure 6:**
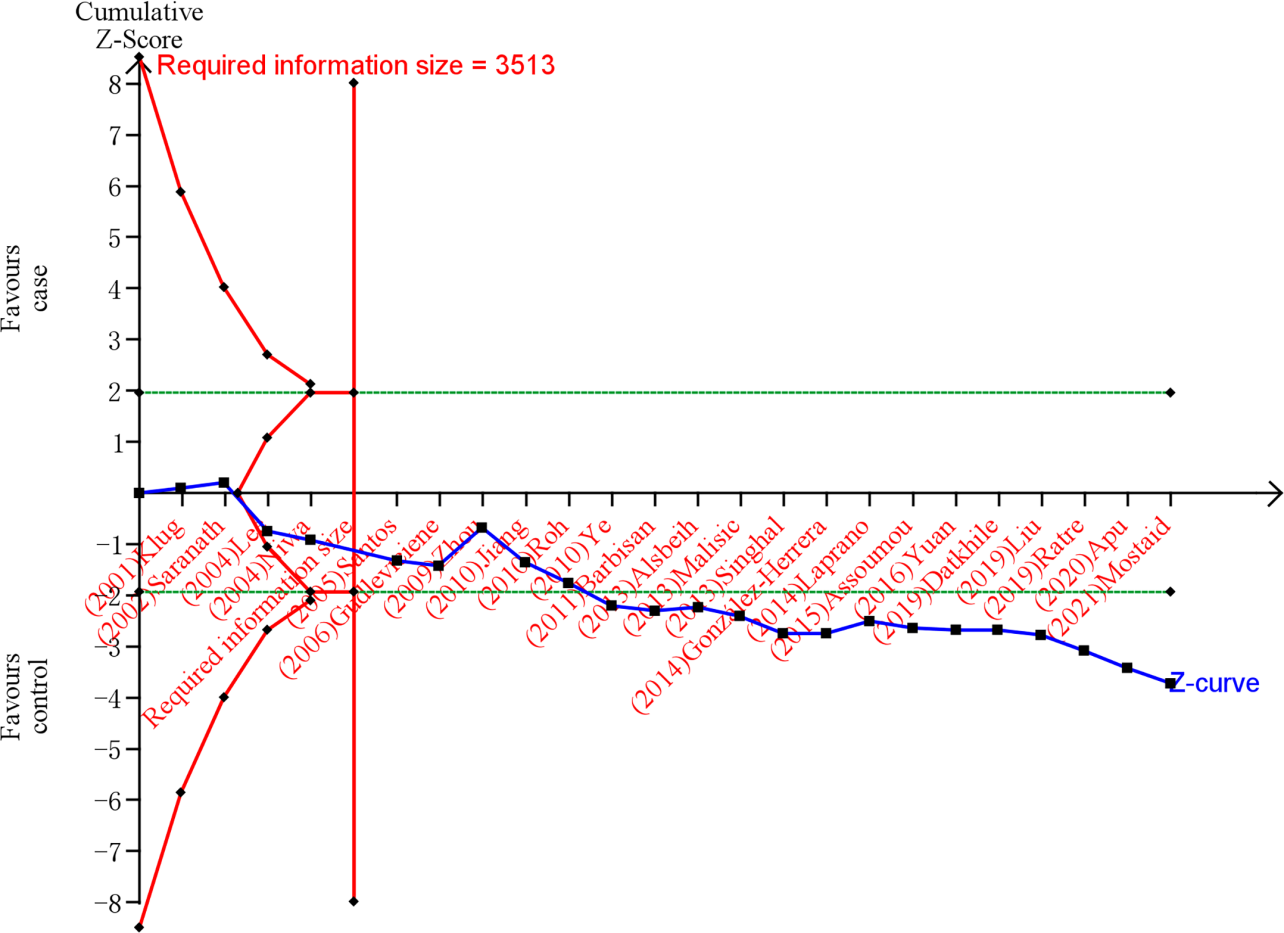
Estimation of the sample size for the relationship between TP53 rs1042522 polymorphism and cervical cancer. Trial sequential analysis (TSA) is a methodology that includes a sample size calculation for a meta-analysis with the threshold of statistical significance. We performed a TSA using an allele model assumption but replaced the allele count with the sample size (divided by 2). Detailed settings: Significance level = 0.05; power = 0.80; ratio of controls to cases = 1; hypothetical proportion of effect allele in control = 0.46; least extreme OR to be detected = 1.3; I^2^ (heterogeneity) = 78%.

After TSA estimation, the results on MDM2 rs2279744 polymorphism showed that the cumulative sample size has exceeded the target sample size (**Figure 5**). Significant relationship was found between MDM2 rs2279744 polymorphism and cervical cancer. Therefore, definite results for the correlation of MDM2 rs2279744 polymorphism and cervical cancer can be obtained. For the relationship between TP53 rs1042522 polymorphism and cervical cancer, the cumulative sample size also exceeded the target sample size (**Figure 6**). There was a significant relationship between TP53 rs1042522 polymorphism and cervical cancer. Thus, definite results could also be obtained.

## Discussion

MDM2 rs2279744 polymorphism was in the MDM2 intronic promoter and shown to cause overexpression of MDM2 RNA and protein (62). A previous study has observed that compared with SNP309 T allele, SNP309 G allele increases MDM2 transcription by prolonging the binding site of transcription factor specificity protein 1 (Sp1) (63). MDM2 rs2279744 polymorphism enhances the binding of transcription factor Sp1 to 309 G allele, which increases MDM2 protein levels by two- to four-fold and reduces TP53 function (64). The increase of MDM2 SNP309T>G leads to the direct inhibition of TP53 transcriptional activity, which makes the damaged cells escape the control of cell-cycle checkpoints and become carcinogenic (22). The p53 tumor-suppressor protein is known as the guardian of human cell anticancer (65). The HPV E6 oncogene has previously been shown to binds to and promotes the degradation of cellular TP53 (66), and the Arg72 variant may be more susceptible to this degradation than the Pro72 variant (67). The association between TP53 rs1042522 polymorphism and cervical cancer was first reported in a study published in 1998, which showed that women who are homozygous for arginine at codon 72 of TP53 gene were seven times more likely to develop cervical cancer than heterozygous women (6). Subsequently, numerous studies have been published on this issue, with widely inconsistent results. Thus, we evaluated the association between MDM2 rs2279744 and TP53 rs1042522 polymorphisms and cervical cancer in the current meta-analysis. The study included 5025 patients with cervical cancer from the 30 eligible articles.

In the current meta-analysis, the MDM2 rs2279744 polymorphism showed a significant correlation with cervical cancer in the recessive model (GG vs GT + TT) and homozygote model (GG vs TT). Women with the GG genotype showed a tendency of higher rate of cervical cancer than those with the GT/TT or TT genotype. MDM2 is the critical negative regulator of TP53 and the dysfunction of these genes might be related to the increase of the cumulative rate of genetic errors, thus promoting the progress of the disease (21). It has been reported that MDM2 rs2279744 polymorphism, a functional T to G mutation in the promoter region, can significantly accelerate tumor formation, indicating that the mutated allele G may be a powerful cancer susceptibility allele (64). Our analysis revealed that women with GG genotype were associated with increased susceptibility to cervical cancer, whereas no correlation was observed in dominant, heterozygote or allele models, suggesting that G allele may be the risk allele for cervical cancer and merely carrying the GG genotype increased the risk of cervical cancer. Subgroup analysis showed that the association between MDM2 rs2279744 polymorphism and cervical cancer was significant only in population-based studies or high-quality studies. However, the finding in overall and subgroup analysis in the recessive model need further validation, because only two studies were included in the recessive model.

Additionally, our finding suggested that cervical cancer was significantly associated with TP53 rs1042522 polymorphism in the dominant model (CC + CG vs GG) and homozygote model (GG vs CC). The pooled analysis of C allele as the effect allele indicated that the risk of cervical cancer in women with CC/CG genotype was 1.759 times higher than that with GG genotype (OR=1.759, 95%CI: 1.186-2.611, P=0.005). however, women carrying GG genotype was 2.452 times more likely to develop cervical cancer than those with the CC genotype in pooled analysis of G allele as the effect allele (OR=2.452, 95%CI: 1.347-4.464, P=0.003). Therefore, our study failed to determine that whether C or G allele was the risk allele. The increased risk of cervical cancer among women with GG genotype in this meta-analysis can be attributed to its susceptibility to degradation by ubiquitin-dependent proteolysis of HPV early oncoprotein (6). Furthermore, the Arg72 form of p53 protein is inefficient in inducing cell cycle arrest, which increases the risk of cancer in people who inherit this allele (68). Based on the significant association between CC/CG genotype and cervical cancer, we can infer that there is an interaction between MDM2 rs2279744 polymorphism and TP53 rs1042522 polymorphism. MDM2 can regulate TP53 by binding to the N-terminal transactivation domain of TP53 to form a negative autoregulatory feedback loop (69). In addition, MDM2 interacts with retinoblastoma tumor suppressor protein (pRB) and binds to the activation domain of E2F transcription factor 1 (E2F1), which inhibits pRB regulatory function (70). Several studies have reported the gene-gene interactions between MDM2 rs2279744 polymorphism and TP53 rs1042522 polymorphism in various cancers (22–24). Moreover, the gene-environment interactions might also influence the singular effect of TP53 rs1042522 polymorphism on cervical cancer.

When stratified by ethnicity, the subgroup analysis suggested a significant association between TP53 rs1042522 polymorphism and cervical cancer in Asians and Europeans, and no significant in other population, which means that TP53 rs1042522 polymorphism may increase the risk of cervical cancer for Asian and European people not others. This difference may be caused by cultural or lifestyle factors and dietary differences between different populations (71). In addition, the correlation of TP53 rs1042522 polymorphisms with cervical cancer was shown only in high-quality studies, suggesting less heterogeneity and relatively stable outcomes between high-quality studies.

Furthermore, the prediction intervals were calculated to evaluate the heterogeneity for the random effects in present meta-analysis. The large 95% prediction intervals in all genetic models suggested a considerably high variation between studies, indicating that the associations of MDM2 rs2279744 and TP53 rs1042522 polymorphisms with cervical cancer risk could be found to be significantly changed in future studies. We speculated that different ethnicity of study population may lead to high between-study heterogeneity. The trim-and-fill analysis showed that there existed publication bias in seven genetic models. And the existence of publication bias changed the results in three models (MDM2 rs2279744: GG vs TT; TP53 rs1042522: CC vs CG + GG and GG vs CC). Correction for potential publication bias did not alter the association in four models (TP53 rs1042522: CG vs GG, C vs G, GG + GC vs CC and GG vs GC + CC). Thus, more relevant studies are needed to verify the significant relationship of MDM2 rs2279744 polymorphism (GG vs TT) or TP53 rs1042522 polymorphism (GG vs CC) and cervical cancer risk.

The present meta-analysis still has several limitations. First, only two studies were included in the recessive models (MDM2: GG vs GT + TT and TP53: GG vs GC + CC), although the pooled result was statistically significant in the recessive model of MDM2 rs2279744 polymorphism, the number of included studies was not convincing enough. Second, Given the small number of studies included, East, South and West Asian were unified into Asians for subgroup analysis. Thus, there are strong differences in terms of genetic background among these populations. Furthermore, the study population in certain countries were from different races, making it difficult to identify the ethnicity of the subjects. Therefore, more included studies are needed to further explored the subgroup analysis conducted by ethnicity. Third, Due to the small number of studies and different ethnicity of study population, there are significant chances of type I error. Although random-effects meta-analyses were conducted for all quantitative synthesis, Numerous studies with large sample need to be included to reduce the possibility of type 1 error. Finally, five studies did not report P values for the Hardy-Weinberg equilibrium in the control group, which may affect the pooled results.

## Conclusion

In summary, this meta-analysis suggested that MDM2 SNP309T>G and TP53 rs1042522 C>G polymorphisms were associated with the increased risk of cervical cancer. Further studies are needed to explore the effect of gene-gene interaction between MDM2 SNP309T>G and TP53 rs1042522 C>G polymorphisms on cervical cancer risk.

## Data availability statement

The original contributions presented in the study are included in the article/**Supplementary Material**. Further inquiries can be directed to the corresponding author.

## Author contributions

MY and XZ conceived and designed the study. MY and QZ searched the literatures and extracted the data. MY and XZ carried out the statistical analyses and data interpretation. MY and QZ wrote the manuscript. All authors contributed to the article and approved the submitted version.

## Conflict of interest

The authors declare that the research was conducted in the absence of any commercial or financial relationships that could be construed as a potential conflict of interest.

## Publisher’s note

All claims expressed in this article are solely those of the authors and do not necessarily represent those of their affiliated organizations, or those of the publisher, the editors and the reviewers. Any product that may be evaluated in this article, or claim that may be made by its manufacturer, is not guaranteed or endorsed by the publisher.
