# Supplementary material for: Associations of MDM2 rs2279744 and TP53 rs1042522 polymorphisms with cervical cancer risk: A meta-analysis and systematic review
Source: Front Oncol. 2022 Aug 19;12:973077. doi: 10.3389/fonc.2022.973077 (PMC9437333; doi:10.3389/fonc.2022.973077)

**FIGURE S1 |** Forest plot of subgroup analysis of TP53 rs1042522 polymorphism and cervical cancer susceptibility in the dominant model (CC + CG vs GG). (A) Subgrouped by ethnicity; (B) subgrouped by source of control; (C) subgrouped by quality score; (D) subgrouped by adjustment.


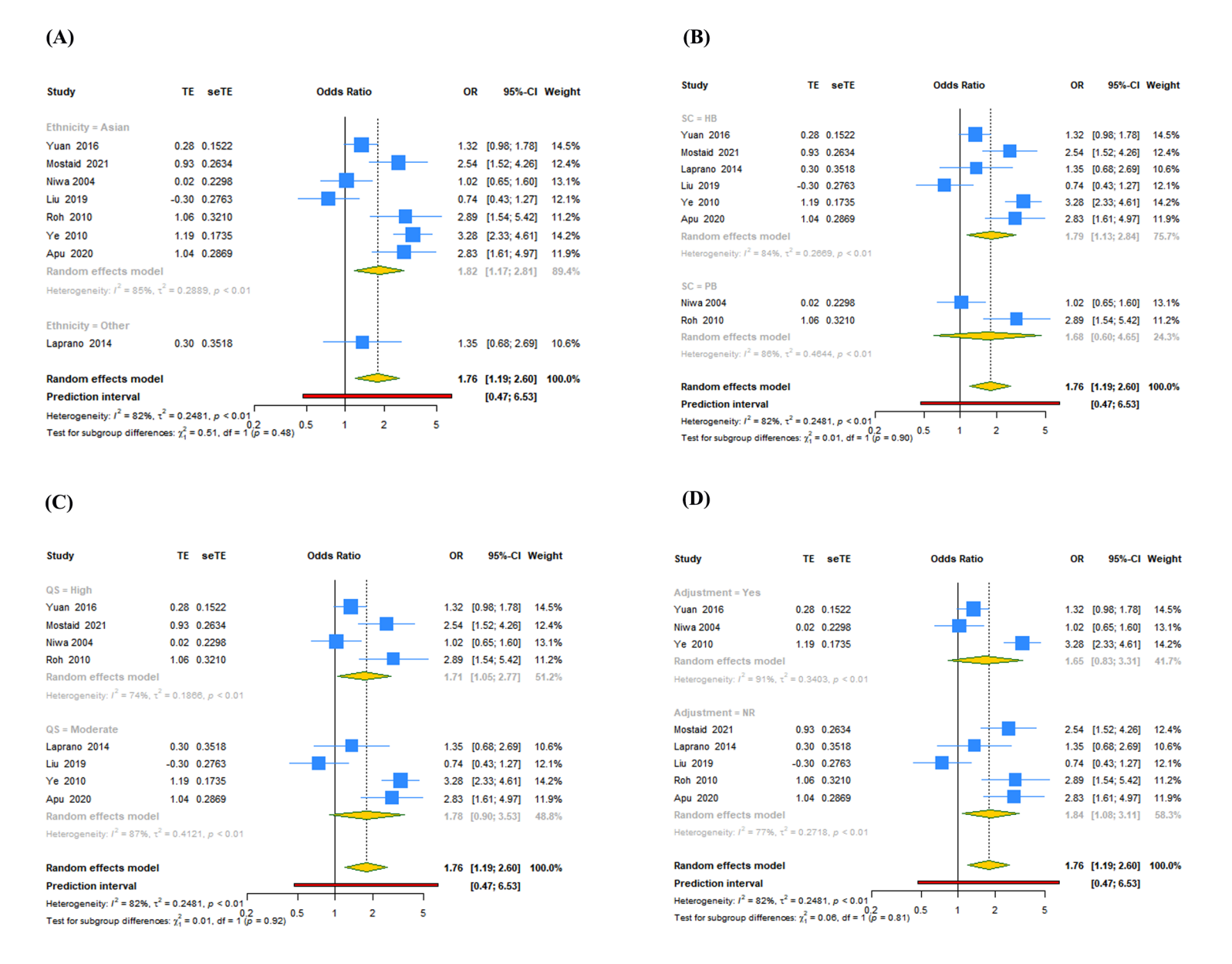


**FIGURE S2 |** Forest plot of subgroup analysis of TP53 rs1042522 polymorphism and cervical cancer susceptibility in the recessive model (CC vs CG + GG). (A) Subgrouped by ethnicity; (B) subgrouped by source of control; (C) subgrouped by quality score; (D) subgrouped by adjustment.


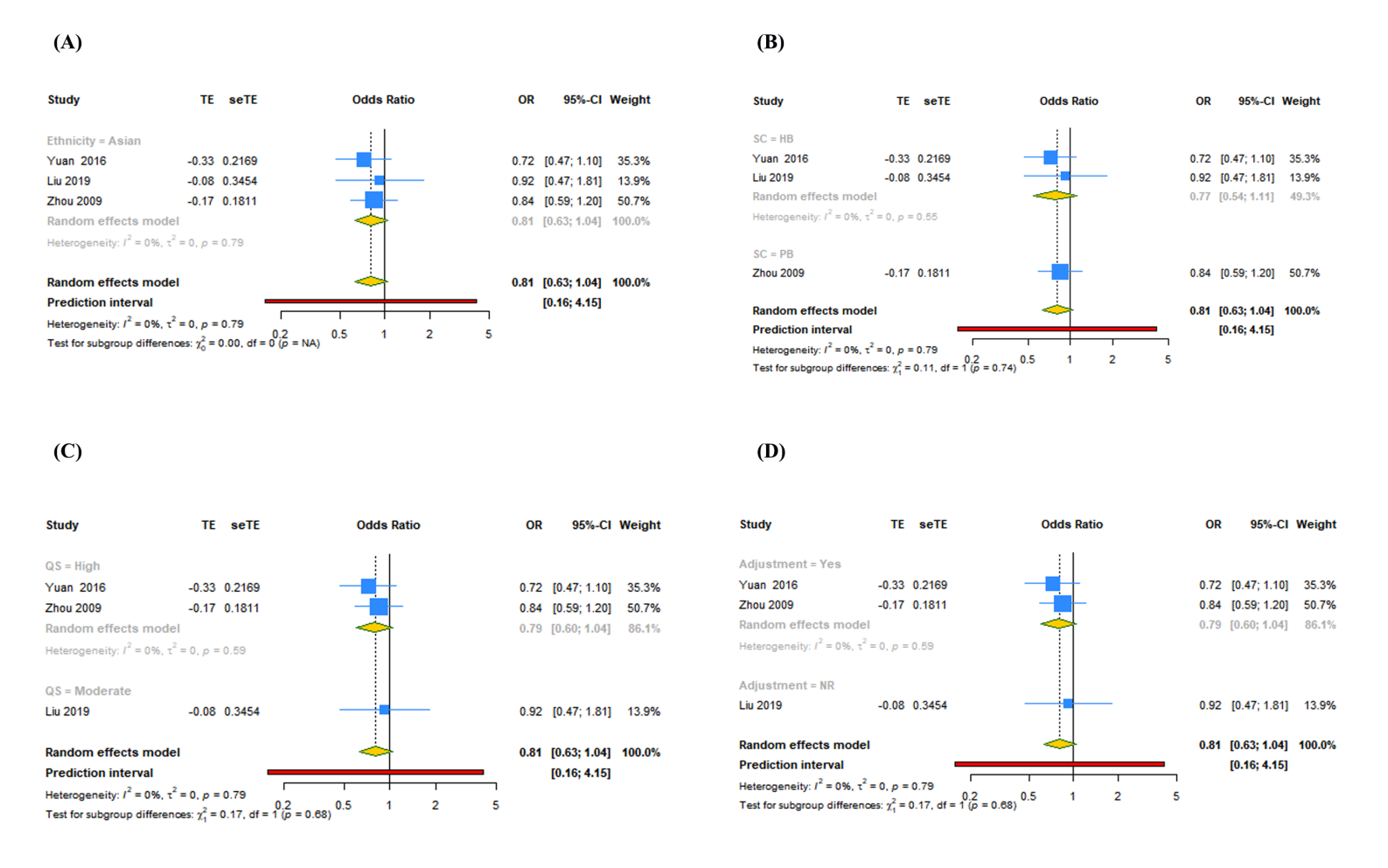


**FIGURE S3 |** Forest plot of subgroup analysis of TP53 rs1042522 polymorphism and cervical cancer susceptibility in the heterozygote model (CG vs GG). (A) Subgrouped by ethnicity; (B) subgrouped by source of control; (C) subgrouped by quality score; (D) subgrouped by adjustment.


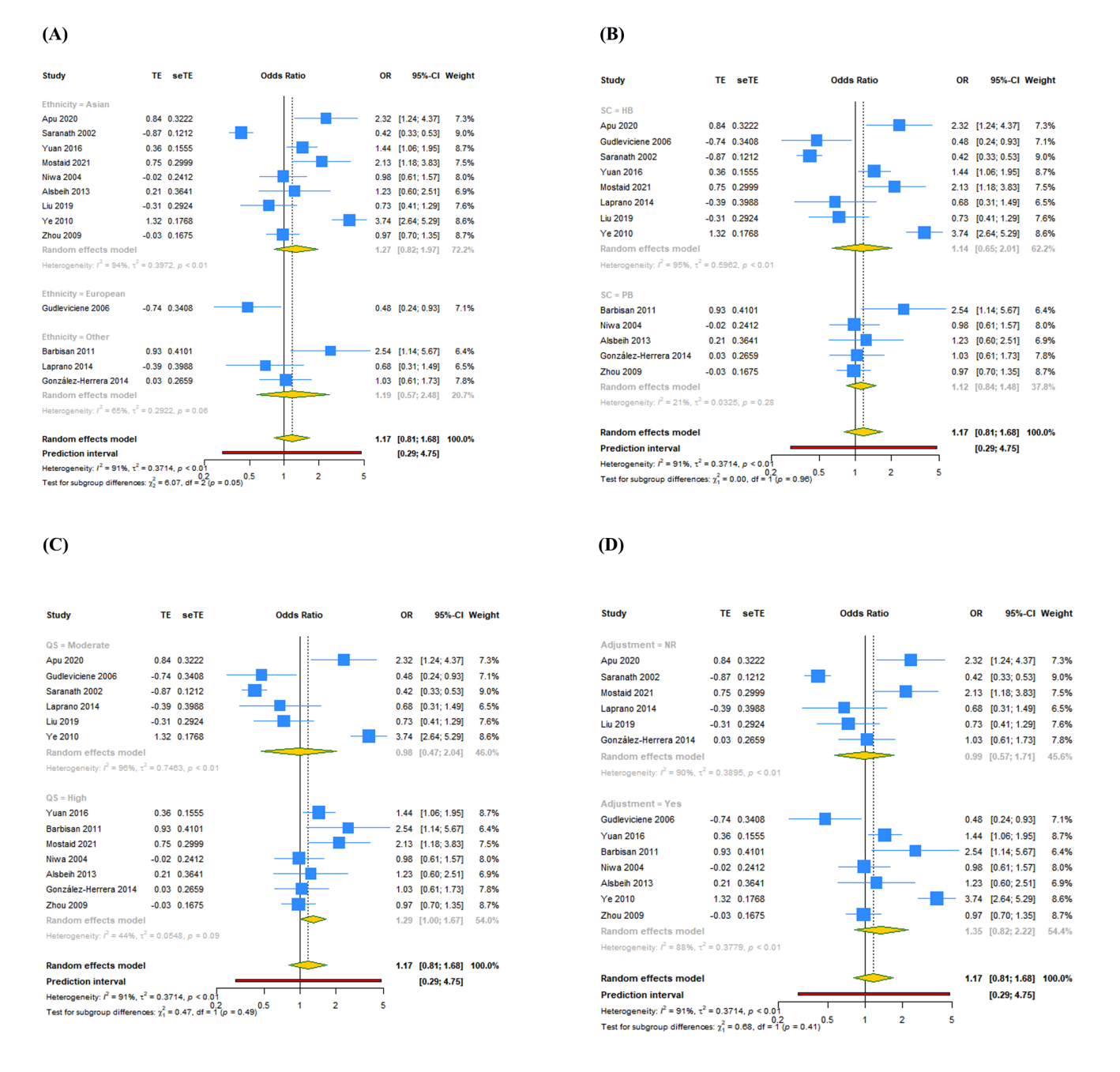


**FIGURE S4 |** Forest plot of subgroup analysis of TP53 rs1042522 polymorphism and cervical cancer susceptibility in the homozygote model (CC vs GG). (A) Subgrouped by ethnicity; (B) subgrouped by source of control; (C) subgrouped by quality score; (D) subgrouped by adjustment.


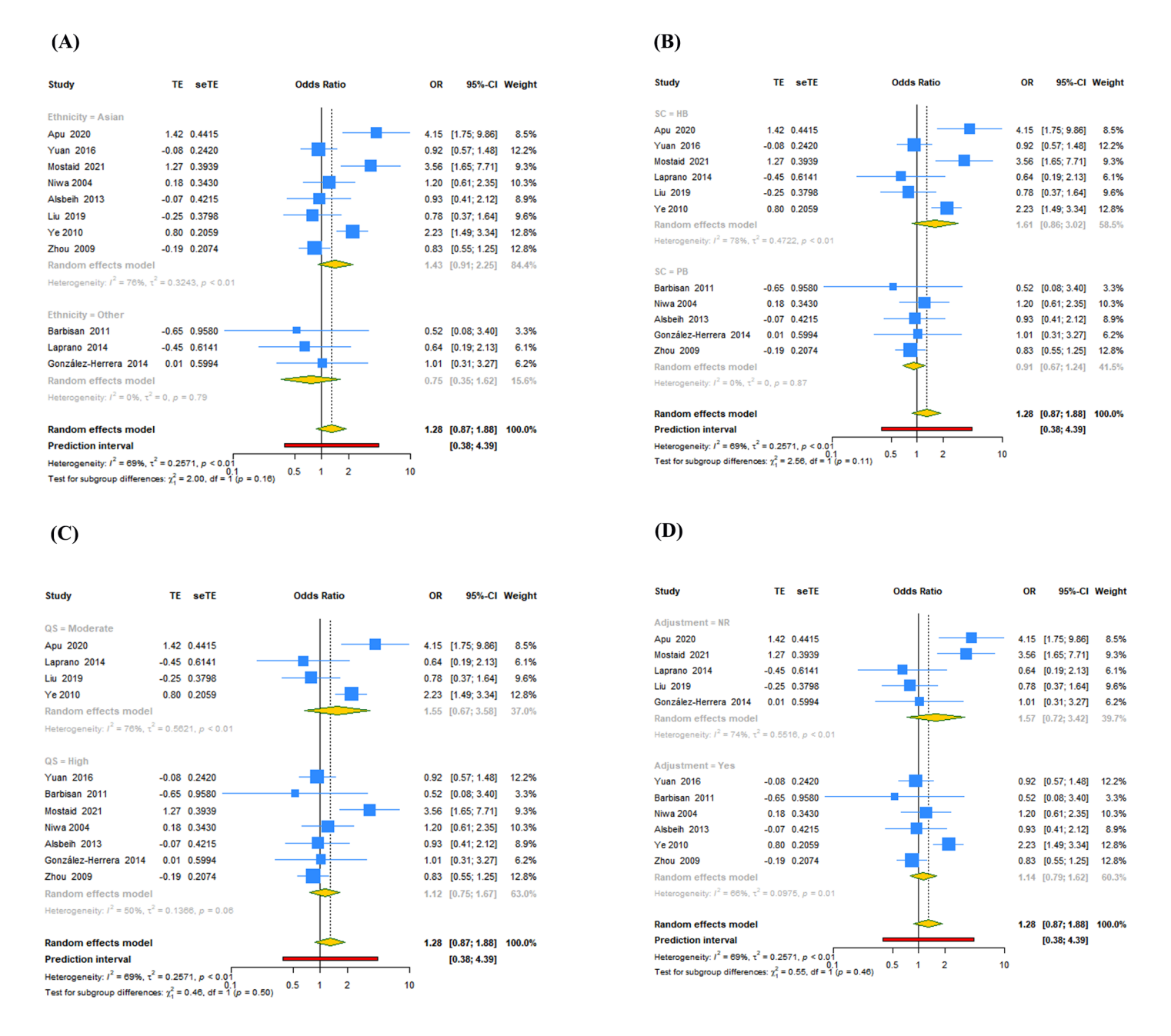


**FIGURE S5 |** Forest plot of subgroup analysis of TP53 rs1042522 polymorphism and cervical cancer susceptibility in the allele model (C vs G). (A) Subgrouped by ethnicity; (B) subgrouped by source of control; (C) subgrouped by quality score; (D) subgrouped by adjustment.


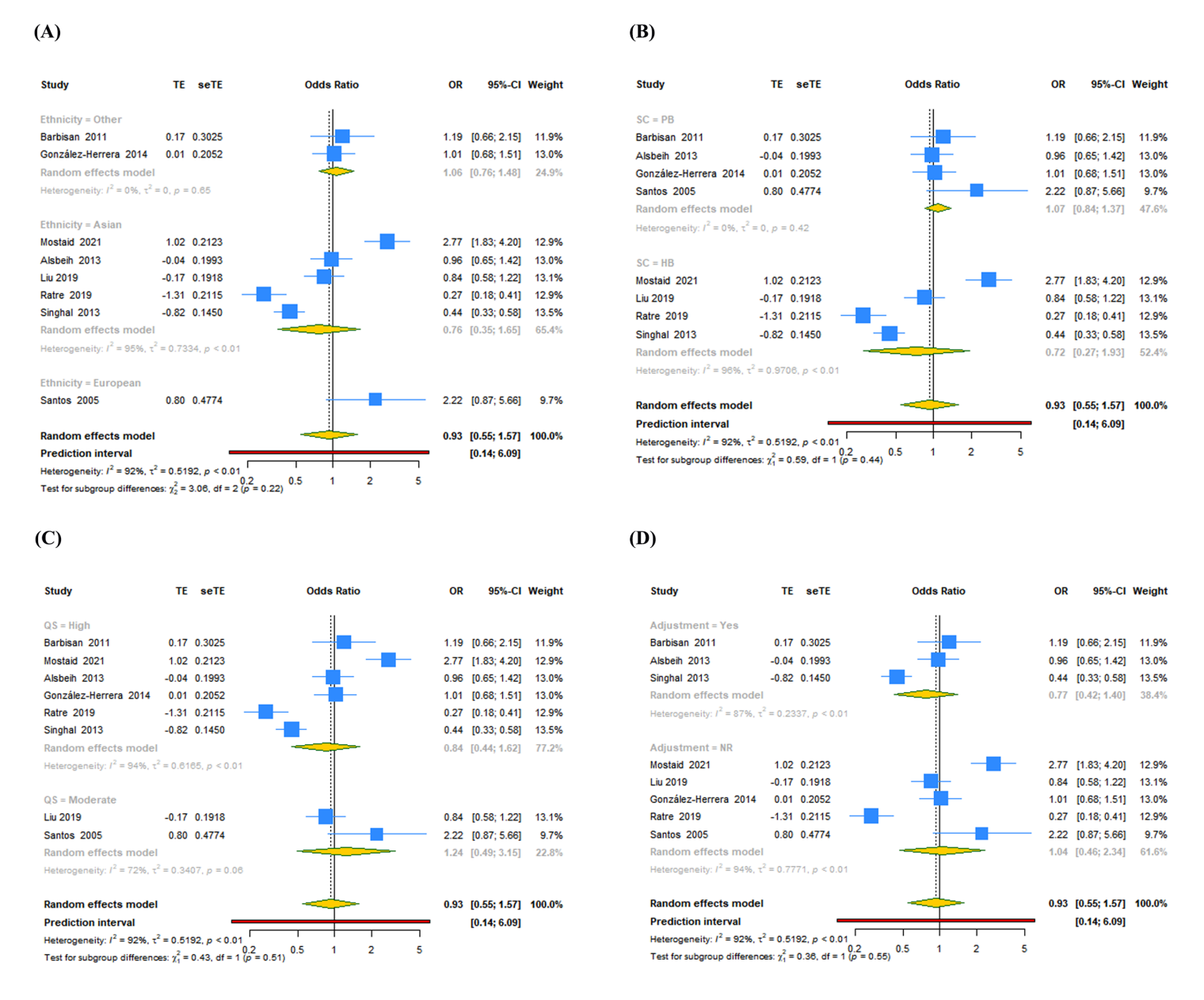


**FIGURE S6 |** Forest plot of subgroup analysis of TP53 rs1042522 polymorphism and cervical cancer susceptibility in the dominant model (GG + GC vs CC). (A) Subgrouped by ethnicity; (B) subgrouped by source of control; (C) subgrouped by quality score; (D) subgrouped by adjustment.


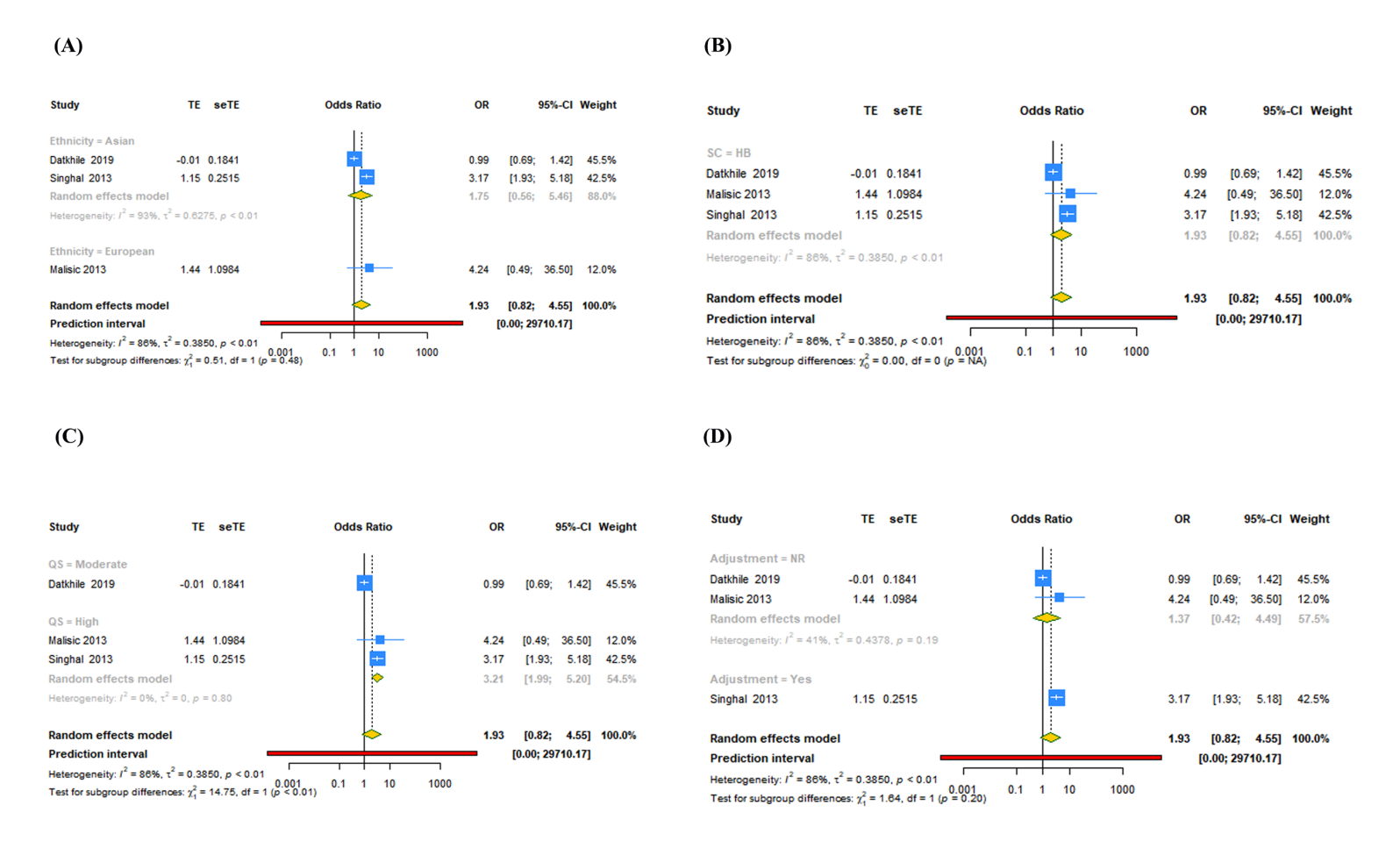


**FIGURE S7 |** Forest plot of subgroup analysis of TP53 rs1042522 polymorphism and cervical cancer susceptibility in the recessive model (GG vs GC + CC). (A) Subgrouped by ethnicity; (B) subgrouped by source of control; (C) subgrouped by quality score; (D) subgrouped by adjustment.


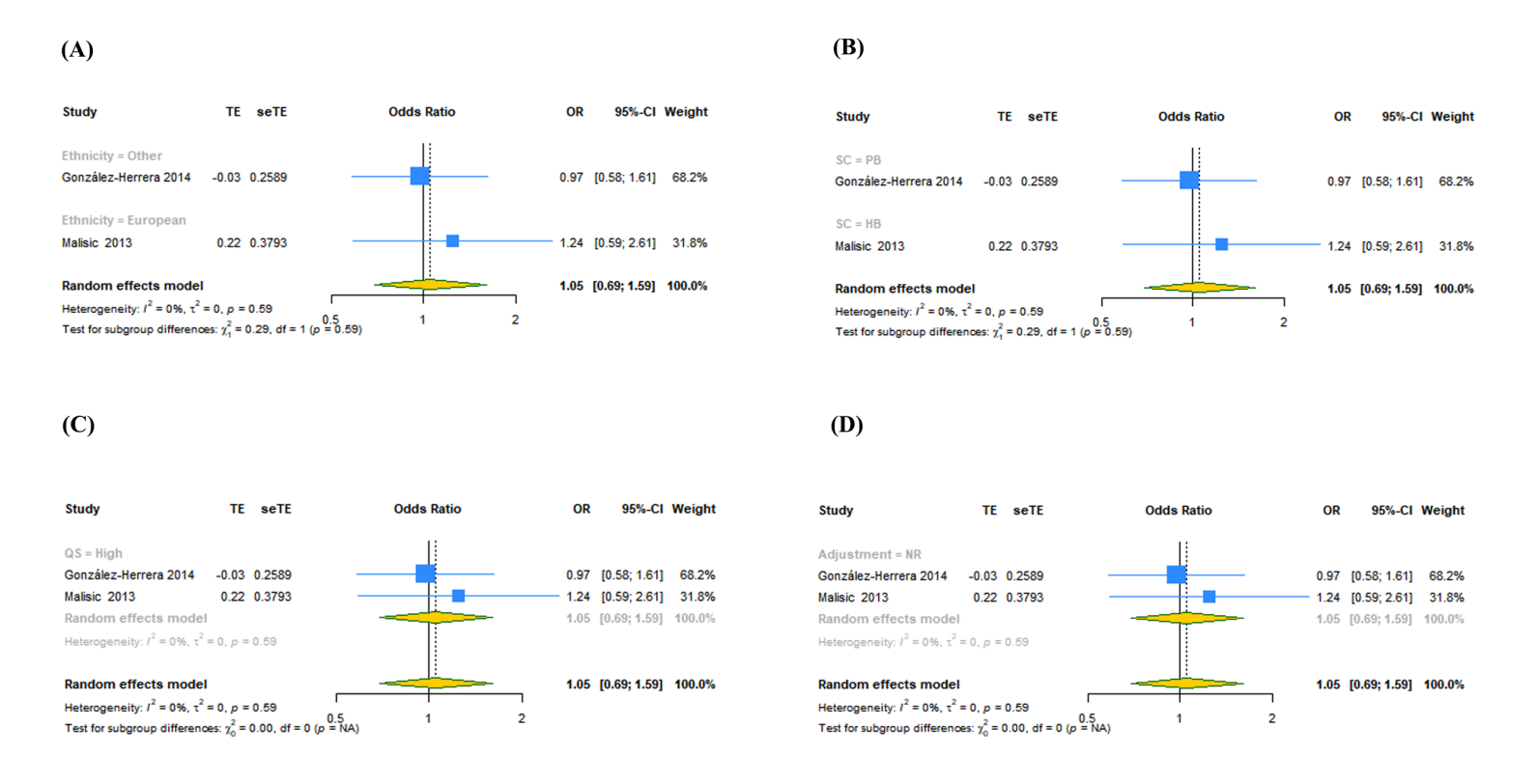


**FIGURE S8 |** Forest plot of subgroup analysis of TP53 rs1042522 polymorphism and cervical cancer susceptibility in the heterozygote model (GC vs CC). (A) Subgrouped by ethnicity; (B) subgrouped by source of control; (C) subgrouped by quality score; (D) subgrouped by adjustment.


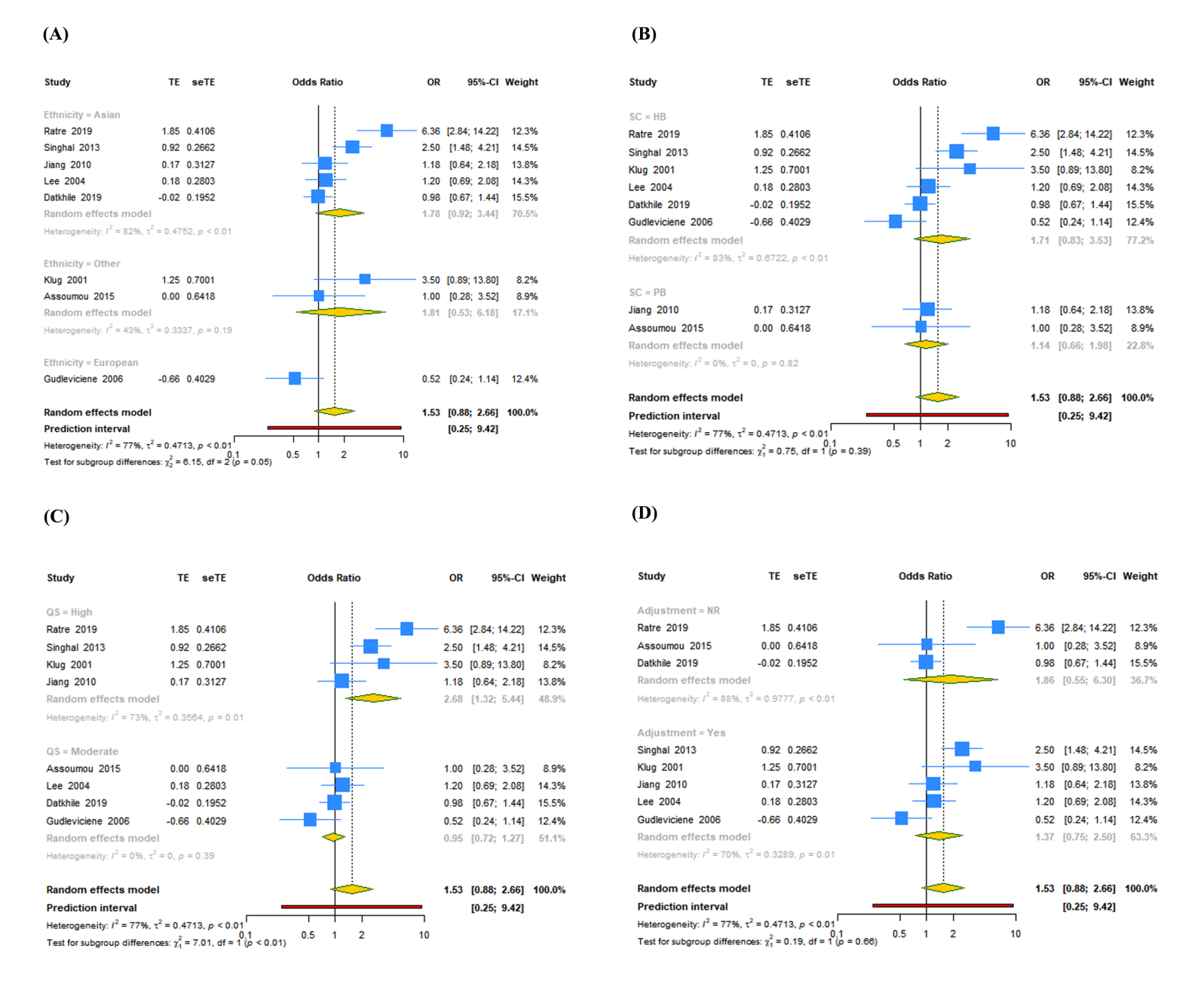


**FIGURE S9 |** Forest plot of subgroup analysis of TP53 rs1042522 polymorphism and cervical cancer susceptibility in the homozygote model (GG vs CC). (A) Subgrouped by ethnicity; (B) subgrouped by source of control; (C) subgrouped by quality score; (D) subgrouped by adjustment.


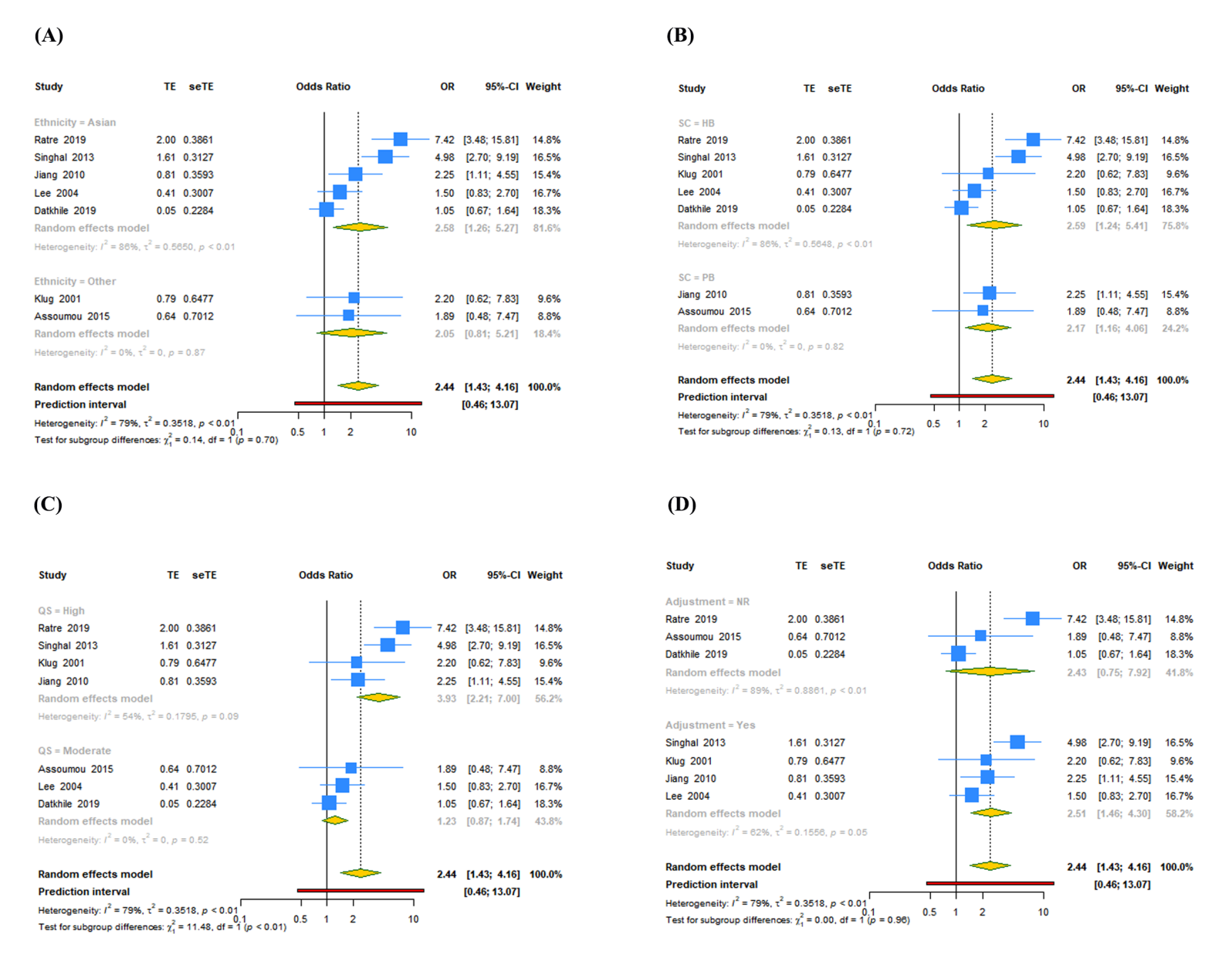

Supplement: Supplementary file 1 [file DataSheet_1.docx]
