# Supplementary material for: Associations of MDM2 rs2279744 and TP53 rs1042522 polymorphisms with cervical cancer risk: A meta-analysis and systematic review
Source: Front Oncol. 2022 Aug 19;12:973077. doi: 10.3389/fonc.2022.973077 (PMC9437333; doi:10.3389/fonc.2022.973077)
Supplement: Supplementary file 3 [file DataSheet_3.docx]

| **TABLE 1 \|** Characteristics of the studies of MDM2 rs2279744 polymorphism included in the meta-analysis. | | | | | | | | | | | | | | | | | | | | | | | | | | | | | |
| --- | --- | --- | --- | --- | --- | --- | --- | --- | --- | --- | --- | --- | --- | --- | --- | --- | --- | --- | --- | --- | --- | --- | --- | --- | --- | --- | --- | --- | --- |
| First author | Year | | Country | | Ethnicity | | Numbers | | | | | Genotyping method | Source of controls | | Genotype | | | | | | EA | | P (HWE) in control | | Adjustments | | | Quality Score | |
|  |  |  |  |  |  |  | Cases | | Controls | | |  |  |  | GG | | GT | | TT | |  |  |  |  |  |  |  |  |  |
| Guo | 2016 | | China | | Asian | | 180 | | 182 | | | PCR-RFLP | PB | | 209 | | 66 | | 87 | | G | | >0.05 | | Age, sex, cigarette smoking, alcohol consumption and family history | | | 7 | |
| Tantengco | 2019 | | Philippine | | Asian | | 28 | | 21 | | | PCR Sequencing | HB | | 5 | | 19 | | 20 | | G | | >0.05 | | NA | | | 6 | |
| Jiang | 2011 | | China | | Asian | | 105 | | 140 | | | PCR-RFLP | HB | | 64 | | 134 | | 47 | | G | | NA | | Age, smoking, drinking and family history | | | 6 | |
| Vargas-Torres | 2014 | | Brazil | | Latino | | 293 | | 184 | | | PCR-RFLP | PB | | 43 | | 186 | | 248 | | G | | 0.644 | | NA | | | 8 | |
| Al-Harbi | 2017 | | Saudi Arabia | | Asian | | 232 | | 313 | | | PCR Sequencing | PB | | 132 | | 260 | | 153 | | G | | 0.885 | | NA | | | 7 | |
| Alsbeih | 2013 | | Saudi Arabia | | Asian | | 100 | | 100 | | | PCR Sequencing | PB | | 53 | | 90 | | 57 | | G | | >0.05 | | Age | | | 7 | |
| Roszak | 2015 | | Poland | | European | | 456 | | 481 | | | PCR primer pairing | HB | | 153 | | 408 | | 376 | | G | | 0.37 | | Age, pregnancy, oral contraceptive use, tobacco smoking, and menopausal status | | | 6 | |
| Singhal | 2013 | | India | | Asian | | 182 | | 182 | | | PCR-RFLP | HB | | 67 | | 126 | | 171 | | G | | >0.05 | | Age and ethnicity | | | 7 | |
| Meissner | 2007 | | Brazil | | Latino | | 70 | | 100 | | | PIRA-PCR assay | PB | | 22 | | 89 | | 61 | | G | | >0.05 | | Age and ethnicity | | | 7 | |
| EA, effect allele; HWE, Hardy-Weinberg equilibrium; PCR-RFLP, polymerase chain reaction-restriction fragment length polymorphism; PB, population-based study; HB, hospital-based study; PIRA, primer-introduced restriction analysis; NA, not available. | | | | | | | | | | | | | | | | | | | | | | | | | | | | | |
| **TABLE 2 \|** Characteristics of the studies of TP53 rs1042522 polymorphism included in the meta-analysis. | | | | | | | | | | | | | | | | | | | | | | | | | | | | |  |
| First author | | Year | | Country | | Ethnicity | | Numbers | | | Genotyping method | | | Source of control | | Genotype | | | | | | EA | | P (HWE) in control | | Adjustments | Quality Score | |  |
|  |  |  |  |  |  |  |  | Cases | | Controls |  |  |  |  |  | CC | | CG | | GG | |  |  |  |  |  |  |  |  |
| Yuan | | 2016 | | China | | Asian | | 328 | | 568 | PCR-RFLP | | | HB | | 116 | | 493 | | 287 | | C | | 0.08 | | Age | 7 | |  |
| Barbisan | | 2011 | | Argentina | | Latino | | 98 | | 123 | PCR Sequencing | | | PB | | 13 | | 97 | | 111 | | C | | >0.05 | | Age and HPV | 7 | |  |
| Mostaid | | 2021 | | Bangladesh | | Asian | | 129 | | 122 | PCR-RFLP | | | HB | | 39 | | 68 | | 144 | | C | | >0.05 | | NA | 7 | |  |
| Laprano | | 2014 | | Brazil | | Latino | | 45 | | 88 | PCR-RFLP | | | HB | | 17 | | 65 | | 51 | | C | | >0.05 | | NA | 6 | |  |
| Niwa | | 2004 | | Japan | | Asian | | 112 | | 442 | PCR-CTPP | | | PB | | 71 | | 261 | | 222 | | C | | 0.92 | | Age | 7 | |  |
| Alsbeih | | 2013 | | Saudi Arabia | | Asian | | 100 | | 100 | PCR Sequencing | | | PB | | 48 | | 110 | | 42 | | C | | 0.72 | | Age | 7 | |  |
| Liu | | 2019 | | China | | Asian | | 121 | | 118 | MAMA-PCR | | | HB | | 87 | | 101 | | 41 | | C | | 0.74 | | NA | 6 | |  |
| González-Herrera | | 2014 | | Mexico | | Latino | | 78 | | 274 | PCR-RFLP | | | PB | | 18 | | 147 | | 187 | | C | | 0.21 | | NA | 8 | |  |
| Roh | | 2010 | | Korea | | Asian | | 53 | | 286 | PCR Sequencing | | | PB | | NA | | NA | | 182 | | C | | >0.05 | | NA | 7 | |  |
| Ye | | 2010 | | China | | Asian | | 500 | | 800 | PCR Sequencing | | | HB | | 279 | | 771 | | 250 | | C | | >0.05 | | Age | 6 | |  |
| Zhou | | 2009 | | China | | Asian | | 404 | | 404 | PCR-RFLP | | | PB | | 163 | | 404 | | 241 | | C | | 0.406 | | Age, smoking status, menopausal status, family history of cancer and parity | 9 | |  |
| Datkhile | | 2019 | | India | | Asian | | 350 | | 400 | PCR-RFLP | | | HB | | 174 | | 394 | | 182 | | G | | NA | | NA | 6 | |  |
| Santos | | 2005 | | Portugal | | European | | 164 | | 145 | AS-PCR | | | PB | | 20 | | 87 | | 202 | | G | | >0.05 | | NA | 6 | |  |
| Malisic | | 2013 | | Serbia | | European | | 49 | | 74 | PCR-RFLP | | | HB | | 7 | | 42 | | 74 | | G | | >0.05 | | NA | 8 | |  |
| Apu | | 2020 | | Bangladesh | | Asian | | 134 | | 102 | PCR-RFLP | | | HB | | 36 | | 62 | | 129 | | C | | >0.05 | | NA | 6 | |  |
| Ratre | | 2019 | | India | | Asian | | 100 | | 100 | PCR-RFLP | | | HB | | 67 | | 59 | | 74 | | G | | >0.05 | | NA | 7 | |  |
| Singhal | | 2013 | | India | | Asian | | 182 | | 182 | PCR-RFLP | | | HB | | 100 | | 170 | | 94 | | G | | >0.05 | | Age, ethnic | 7 | |  |
| Klug | | 2001 | | Peru | | Latino | | 119 | | 127 | PCR-RFLP | | | HB | | 30 | | 90 | | 126 | | G | | >0.05 | | Age and HPV | 7 | |  |
| Jiang | | 2010 | | China | | Asian | | 104 | | 160 | PCR-RFLP | | | PB | | 70 | | 131 | | 63 | | G | | >0.05 | | Age, cigarette smoking, alcohol consumption and family history | 8 | |  |
| Assoumou | | 2015 | | Gabon | | African | | 31 | | 71 | PCR Sequencing | | | PB | | 15 | | 60 | | 27 | | G | | >0.05 | | NA | 5 | |  |
| Gudleviciene | | 2006 | | Lithuania | | European | | 141 | | 97 | PCR-RFLP | | | HB | | 35 | | 149 | | 54 | | G | | NA | | Age | 6 | |  |
| Saranath | | 2002 | | India | | Asian | | 134 | | 131 | PCR Sequencing | | | HB | | 53 | | 165 | | 47 | | G | | NA | | NA | 6 | |  |
| Lee | | 2004 | | Korea | | Asian | | 185 | | 345 | SNaPshot assay | | | HB | | 84 | | 242 | | 204 | | G | | NA | | Age, education level, age at first intercourse, and number of children | 4 | |  |
| EA, effect allele; HWE, Hardy-Weinberg equilibrium; PCR-RFLP, polymerase chain reaction-restriction fragment length polymorphism; HB, hospital-based study; PB, population-based study; PCR-CTPP, PCR with confronting two-pair primers; MAMA-PCR, mismatch amplification mutation assay PCR; AS-PCR, allele-specific polymerase chain reaction; NA, not available. | | | | | | | | | | | | | | | | | | | | | | | | | | | | |  |

| **TABLE 3 \|** The association between MDM2 rs2279744 polymorphism and cervical cancer susceptibility. | | | | | | | | | | | | | | | | | | | | |
| --- | --- | --- | --- | --- | --- | --- | --- | --- | --- | --- | --- | --- | --- | --- | --- | --- | --- | --- | --- | --- |
| Outcome and subgroups | Dominant model (GG + GT vs TT) | | | | Recessive model (GG vs GT + TT) | | | | Heterozygote model (GT vs TT) | | | | Homozygote model (GG vs TT) | | | | Allele model (G vs T) | | | |
|  | No | OR (95% CI) | I^2^, % | 95% PI | No | OR (95% CI) | I^2^, % | 95% PI | No | OR (95% CI) | I^2^, % | 95% PI | No | OR (95% CI) | I^2^, % | 95% PI | No | OR (95% CI) | I^2^, % | 95% PI |
| Overall | 5 | 1.393 (0.987-1.968) | 70.2 | 0.429-4.522 | 2 | **1.602 (1.077-2.383)** | 0.0 | - | 8 | 1.140 (0.751-1.729) | 66.1 | 0.291-4.465 | 8 | **1.469 (1.031-2.095)** | 65.7 | 0.516-4.184 | 7 | 1.246 (0.927-1.676) | 77.2 | 0.472-3.294 |
| Ethnicity | | | | | | | | | | | | | | | | | | | | |
| Asian | 2 | 1.815 (0.812-4.057) | 86.7 | - | 1 | **1.657 (1.016-2.703)** | - | - | 6 | 1.079 (0.576-2.021) | 75.7 | 0.130-8.985 | 6 | 1.578 (0.969-2.569) | 67.9 | 0.361-6.902 | 6 | 1.238 (0.865-1.772) | 81.0 | 0.370-4.145 |
| European | 1 | 1.180 (0.907-1.535) | - | - |  |  |  |  | 1 | 1.174 (0.876-1.573) | - | - | 1 | 1.099 (0.910-1.328) | - | - |  |  |  |  |
| Other | 2 | 1.192 (0.856-1.661) | 0.0 | - | 1 | 1.500 (0.760-2.960) | - | - | 1 | 1.180 (0.798-1.745) | - | - | 1 | 1.610 (0.800-3.240) | - | - | 1 | 1.240 (0.925-1.662) | - | - |
| Source of control | | | | | | | | | | | | | | | | | | | | |
| PB | 3 | 1.196 (0.923-1.551) | 0.0 | 0.222-6.443 | 2 | **1.602 (1.077-2.383)** | 0.0 | - | 4 | 1.109 (0.802-1.534) | 36.7 | 0.353-3.481 | 4 | 1.208 (0.864-1.687) | 22.0 | 0.435-3.352 | 4 | 1.103 (0.906-1.343) | 38.3 | 0.547-2.225 |
| HB | 2 | 1.768 (0.777-4.026) | 91.2 | - |  |  |  |  | 4 | 1.002 (0.376-2.668) | 79.4 | 0.011-90.327 | 4 | **1.902 (1.048-3.451)** | 81.8 | 0.173-20.970 | 3 | 1.418 (0.670-2.998) | 76.5 | - |
| Quality score | | | | | | | | | | | | | | | | | | | | |
| High | 4 | 1.464 (0.939-2.283) | 73.7 | 0.218-9.816 | 2 | **1.602 (1.077-2.383)** | 0.0 | - | 5 | 1.328 (0.887-1.988) | 70.8 | 0.337-5.235 | 5 | 1.482 (0.895-2.454) | 70.7 | 0.258-8.504 | 5 | 1.270 (0.914-1.766) | 81.9 | 0.379-4.259 |
| Moderate | 1 | 1.180 (0.907-1.535) | - | - |  |  |  |  | 3 | 0.727 (0.249-2.124) | 66.7 | - | 3 | 1.403 (0.816-2.412) | 54.6 | 0.006-319.575 | 2 | 1.054 (0.399-2.785) | 74.8 | - |
| Adjustment | | | | | | | | | | | | | | | | | | | | |
| Yes | 4 | 1.431 (0.914-2.240) | 77.2 | 0.203-10.066 | 1 | **1.657 (1.016-2.703)** | - | - | 5 | 1.420 (0.995-2.026) | 58.2 | 0.468-4.310 | 5 | 1.612 (0.949-2.741) | 79.1 | 0.243-10.688 | 4 | 1.446 (0.970-2.157) | 81.0 | 0.236-8.856 |
| NR | 1 | 1.250 (0.867-1.803) | - | - | 1 | 1.500 (0.760-2.960) | - | - | 3 | 0.684 (0.251-1.866) | 68.9 | - | 3 | 1.167 (0.790-1.723) | 0.0 | 0.093-14.599 | 3 | 1.039 (0.777-1.391) | 34.2 | 0.057-18.817 |

| **TABLE 4 \|** The association between TP53 rs1042522 polymorphism and cervical cancer susceptibility (allele C as the effect allele). | | | | | | | | | | | | | | | | | | | | |
| --- | --- | --- | --- | --- | --- | --- | --- | --- | --- | --- | --- | --- | --- | --- | --- | --- | --- | --- | --- | --- |
| Outcome and subgroups | Dominant model (CC + CG vs GG) | | | | Recessive model (CC vs CG + GG) | | | | Heterozygote model (CG vs GG) | | | | Homozygote model (CC vs GG) | | | | Allele model (C vs G) | | | |
|  | No | OR (95% CI) | I2, % | 95% PI | No | OR (95% CI) | I2, % | 95% PI | No | OR (95% CI) | I2, % | 95% PI | No | OR (95% CI) | I2, % | 95% PI | No | OR (95% CI) | I2, % | 95% PI |
| Overall | 8 | **1.759 (1.192-2.596)** | 82.3 | 0.474-6.533 | 3 | 0.806 (0.626-1.037) | 0.0 | 0.157-4.148 | 13 | 1.168 (0.811-1.681) | 91.3 | 0.287-4.747 | 11 | 1.283 (0.874-1.885) | 68.7 | 0.375-4.389 | 8 | 0.927 (0.546-1.572) | 91.9 | 0.141-6.089 |
| Ethnicity | | | | | | | | | | | | | | | | | | | | |
| Asian | 7 | **1.817 (1.172-2.814)** | 84.6 | 0.407-8.110 | 3 | 0.806 (0.626-1.037) | 0.0 | 0.157-4.148 | 9 | 1.268 (0.815-1.972) | 93.7 | 0.261-6.173 | 8 | 1.429 (0.906-2.255) | 76.2 | 0.317-6.438 | 5 | 0.765 (0.354-1.651) | 94.7 | 0.038-15.336 |
| European |  |  |  |  |  |  |  |  | 1 | **0.476 (0.244-0.928)** | - | - |  |  |  |  | 1 | 2.222 (0.872-5.664) | - | - |
| Other | 1 | 1.350 (0.678-2.690) | - | - |  |  |  |  | 3 | 1.192 (0.572-2.481) | 64.5 | - | 3 | 0.751 (0.349-1.618) | 0.0 | 0.005-108.670 | 2 | 1.064 (0.763-1.484) | 0.0 | - |
| Source of control | | | | | | | | | | | | | | | | | | | | |
| PB | 2 | 1.676 (0.605-4.646) | 85.6 | - | 1 | 0.840 (0.589-1.198) | - | - | 5 | 1.120 (0.845-1.485) | 21.3 | 0.537-2.333 | 5 | 0.910 (0.671-1.235) | 0.0 | 0.555-1.494 | 4 | 1.075 (0.841-1.372) | 0.0 | 0.628-1.838 |
| HB | 6 | **1.795 (1.133-2.844)** | 84.0 | 0.371-8.675 | 2 | 0.772 (0.538-1.106) | 0.0 | - | 8 | 1.139 (0.646-2.009) | 94.7 | 0.151-8.565 | 6 | 1.610 (0.859-3.020) | 77.7 | 0.196-13.223 | 4 | 0.724 (0.271-1.935) | 95.9 | 0.006-84.228 |
| Quality Score | | | | | | | | | | | | | | | | | | | | |
| High | 4 | **1.707 (1.050-2.773)** | 74.3 | 0.200-14.539 | 2 | 0.789 (0.600-1.035) | 0.0 | - | 7 | 1.289 (0.997-1.667) | 44.4 | 0.647-2.570 | 7 | 1.122 (0.753-1.671) | 49.9 | 0.379-3.317 | 6 | 0.845 (0.440-1.621) | 93.8 | 0.079-9.014 |
| Moderate | 4 | 1.782 (0.899-3.533) | 87.2 | 0.077-41.354 | 1 | 0.920 (0.468-1.810) | - | - | 6 | 0.983 (0.474-2.036) | 95.8 | 0.072-13.377 | 4 | 1.547 (0.668-3.580) | 75.7 | 0.038-63.498 | 2 | 1.238 (0.487-3.150) | 72.0 | - |
| Adjustment | | | | | | | | | | | | | | | | | | | | |
| Yes | 3 | 1.654 (0.827-3.307) | 90.9 | - | 2 | 0.789 (0.600-1.035) | 0.0 | - | 7 | 1.347 (0.819-2.216) | 88.0 | 0.244-7.447 | 6 | 1.136 (0.794-1.625) | 65.6 | 0.416-3.101 | 3 | 0.766 (0.420-1.396) | 86.7 | - |
| NR | 5 | **1.836 (1.084-3.110)** | 77.0 | 0.284-11.875 | 1 | 0.920 (0.468-1.810) | - | - | 6 | 0.986 (0.569-1.709) | 89.5 | 0.147-5.590 | 5 | 1.572 (0.723-3.417) | 73.5 | 0.108-22.898 | 5 | 1.042 (0.464-2.340) | 93.8 | 0.047-23.079 |

| **TABLE 5 \|** The association between TP53 rs1042522 polymorphism and cervical cancer susceptibility (allele G as the effect allele). | | | | | | | | | | | | | | | | |
| --- | --- | --- | --- | --- | --- | --- | --- | --- | --- | --- | --- | --- | --- | --- | --- | --- |
| Outcome and subgroups | Dominant model (GG + GC vs CC) | | | | Recessive model (GG vs GC + CC) | | | | Heterozygote model (GC vs CC) | | | | Homozygote model (GG vs CC) | | | |
|  | No | OR (95% CI) | I^2^, % | 95% PI | No | OR (95% CI) | I^2^, % | 95% PI | No | OR (95% CI) | I^2^, % | 95% PI | No | OR (95% CI) | I^2^, % | 95% PI |
| Overall | 3 | 1.932 (0.821-4.547) | 86.5 | - | 2 | 1.049 (0.690-1.595) | 0.0 | - | 8 | 1.534 (0.885-2.658) | 76.9 | 0.250-9.416 | 7 | **2.442 (1.433-4.162)** | 79.2 | 0.456-13.071 |
| Ethnicity | | | | | | | | | | | | | | | | |
| Asian | 2 | 1.749 (0.560-5.463) | 92.8 | - |  |  |  |  | 5 | 1.782 (0.923-3.439) | 81.8 | 0.155-20.436 | 5 | **2.576 (1.259-5.271)** | 86.1 | 0.180-36.817 |
| European | 1 | 4.240 (0.493-36.503) | - | - | 1 | 1.240 (0.590-2.608) | - | - | 1 | 0.518 (0.235-1.141) | - | - |  |  |  |  |
| Other |  |  |  |  | 1 | 0.970 (0.584-1.611) | - | - | 2 | 1.813 (0.532-6.180) | 42.5 | - | 2 | 2.051 (0.807-5.213) | 0.0 | - |
| Source of control | | | | | | | | | | | | | | | | |
| PB |  |  |  |  | 1 | 0.970 (0.584-1.611) | - | - | 2 | 1.143 (0.659-1.983) | 0.0 | - | 2 | **2.170 (1.159-4.061)** | 0.0 | - |
| HB | 3 | 1.932 (0.821-4.547) | 86.5 | - | 1 | 1.240 (0.590-2.608) | - | - | 6 | 1.711 (0.830-3.528) | 83.2 | 0.141-20.771 | 5 | **2.589 (1.239-5.410)** | 86.1 | 0.179-37.547 |
| Quality Score | | | | | | | | | | | | | | | | |
| High | 2 | **3.213 (1.987-5.196)** | 0.0 | - | 2 | 1.049 (0.690-1.595) | 0 | - | 4 | **2.677 (1.317-5.444)** | 73.0 | 0.133-53.997 | 4 | **3.934 (2.210-7.001)** | 53.5 | 0.428-36.195 |
| Moderate | 1 | 0.990 (0.690-1.420) | - | - |  |  |  |  | 4 | 0.954 (0.718-1.267) | 0.0 | 0.511-1.779 | 3 | 1.231 (0.872-1.739) | 0.0 | 0.132-11.528 |
| Adjustment | | | | | | | | | | | | | | | | |
| Yes | 1 | **3.167 (1.934-5.185)** | - | - |  |  |  |  | 5 | 1.369 (0.749-2.503) | 69.6 | 0.173-10.866 | 4 | **2.509 (1.465-4.298)** | 61.8 | 0.317-19.843 |
| NR | 2 | 1.369 (0.418-4.485) | 41.4 | - | 2 | 1.049 (0.690-1.595) | 0 | - | 3 | 1.857 (0.548-6.295) | 88.3 | - | 3 | 2.431 (0.747-7.915) | 89.5 | - |

| **TABLE 6 \|** Comparison of pooled effects before and after trim-and-fill analysis. | | | | |
| --- | --- | --- | --- | --- |
| Genetic models | Before trim-and-fill analysis | | After trim-and-fill analysis | |
|  | OR | 95% CI | OR | 95% CI |
| MDM2 rs2279744 | | | | |
| GG + GT vs TT | 1.393 | 0.987-1.968 | 1.393 | 0.987-1.968 |
| GG vs GT + TT | 1.602 | 1.077-2.383 | 1.602 | 1.077-2.383 |
| GT vs TT | 1.140 | 0.751-1.729 | 1.140 | 0.751-1.729 |
| GG vs TT | 1.469 | 1.031-2.095 | **1.149** | **0.785-1.684** |
| G vs T | 1.246 | 0.927-1.676 | 1.246 | 0.927-1.676 |
| TP53 rs1042522 | | | | |
| CC + CG vs GG | 1.759 | 1.192-2.596 | 1.759 | 1.192-2.596 |
| CC vs CG + GG | 0.806 | 0.626-1.037 | **0.788** | **0.622-0.999** |
| CG vs GG | 1.168 | 0.811-1.681 | 0.665 | 0.413-1.072 |
| CC vs GG | 1.283 | 0.874-1.885 | 1.283 | 0.874-1.885 |
| C vs G | 0.927 | 0.546-1.572 | 0.614 | 0.355-1.063 |
| GG + GC vs CC | 1.932 | 0.821-4.547 | 0.990 | 0.355-2.759 |
| GG vs GC + CC | 1.049 | 0.690-1.595 | 0.970 | 0.673-1.397 |
| GC vs CC | 1.534 | 0.885-2.658 | 1.534 | 0.885-2.658 |
| GG vs CC | 2.442 | 1.433-4.162 | **1.275** | **0.656-2.478** |
