# Supplementary material for: Associations of MDM2 rs2279744 and TP53 rs1042522 polymorphisms with cervical cancer risk: A meta-analysis and systematic review
Source: Front Oncol. 2022 Aug 19;12:973077. doi: 10.3389/fonc.2022.973077 (PMC9437333; doi:10.3389/fonc.2022.973077)

**FIGURE S1 |** Sensitivity analysis on the relationship between MDM2 rs2279744 polymorphism and cervical cancer susceptibility in five models. (A) GG + GT vs TT; (B) GG vs GT + TT; (C) GT vs TT; (D) GG vs TT; (E) G vs T.


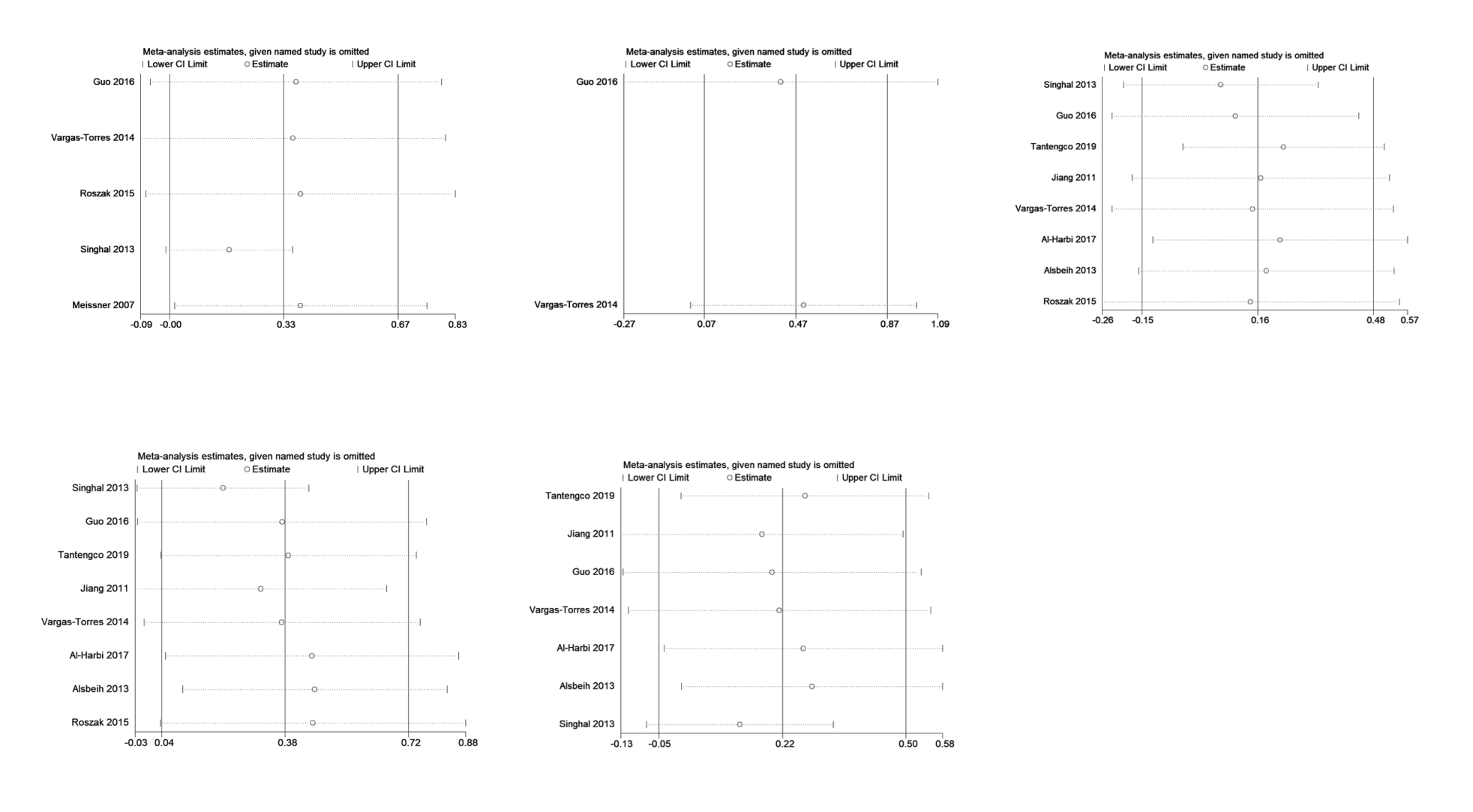


**FIGURE S2 |** Sensitivity analysis on the relationship between TP53 rs1042522 polymorphism and cervical cancer susceptibility in five models (allele C as the effect allele). (A) CC + CG vs GG; (B) CC vs CG + GG; (C) CG vs GG; (D) CC vs GG; (E) C vs G.


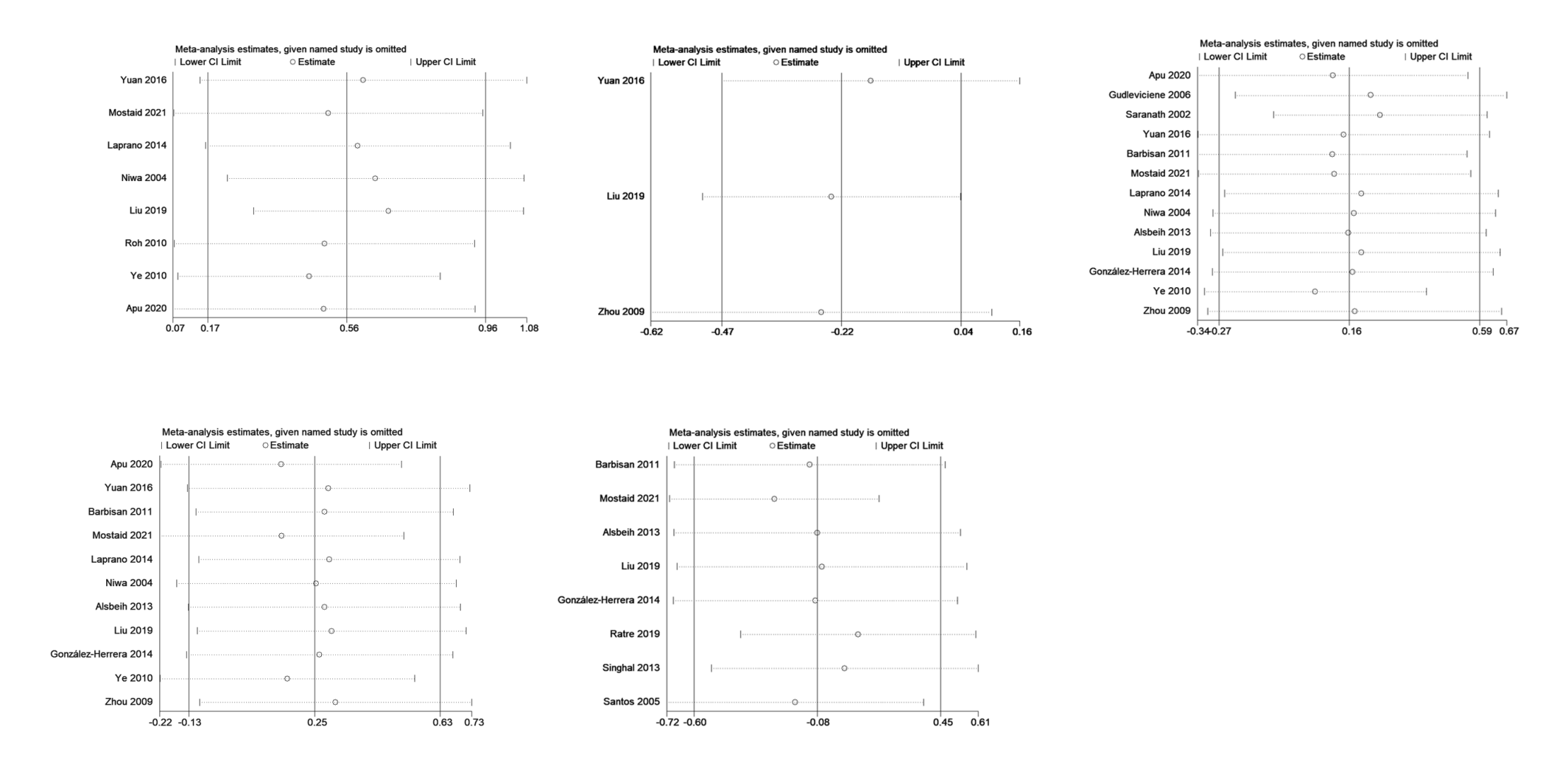


**FIGURE S3 |** Sensitivity analysis on the relationship between TP53 rs1042522 polymorphism and cervical cancer susceptibility in four models (allele G as the effect allele). (A) GG + GC vs CC; (B) GG vs GC + CC; (C) GC vs CC; (D) GG vs CC.


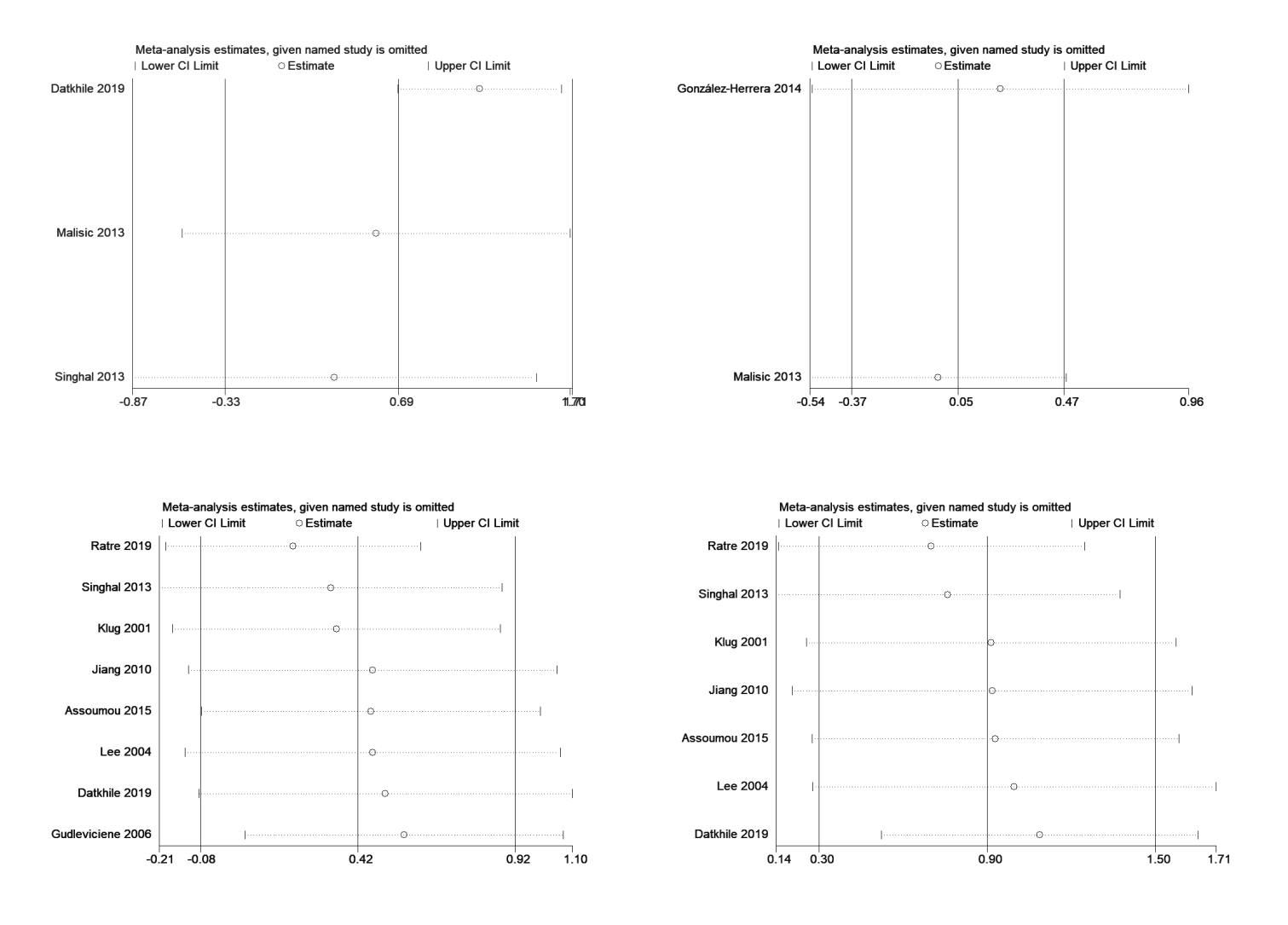

Supplement: Supplementary file 4 [file DataSheet_4.docx]
